# Predictors of fluid responsiveness in critically ill patients mechanically ventilated at low tidal volumes: systematic review and meta-analysis

**DOI:** 10.1186/s13613-021-00817-5

**Published:** 2021-02-08

**Authors:** Jorge Iván Alvarado Sánchez, Juan Daniel Caicedo Ruiz, Juan José Diaztagle Fernández, William Fernando Amaya Zuñiga, Gustavo Adolfo Ospina-Tascón, Luis Eduardo Cruz Martínez

**Affiliations:** 1 Department of Anaesthesiology, Centro Policlínico del Olaya, Bogotá, Colombia; 2 Department of Physiology Sciences, Faculty of Medicine, Universidad Nacional de Colombia, Bogotá, Colombia; 3 Fundación Universitaria de Ciencias de La Salud, Bogotá, Colombia; 4 Department of Internal Medicine, Hospital de San José, Bogotá, Colombia; 5 Department of Anaesthesiology, Fundación Santa Fe de Bogotá, Bogotá, Colombia; 6 Department of Intensive Care, Fundación Valle del Lili - Universidad ICESI, Cali, Colombia

**Keywords:** Pulse pressure variation, Stroke volume variation, End-expiratory occlusion test, Passive leg raising, Tidal volume challenge, Inferior vena cava respiratory variability, Mini-fluid challenge, Fluid responsiveness, Critical care, Critical illness

## Abstract

**Introduction:**

Dynamic predictors of fluid responsiveness have shown good performance in mechanically ventilated patients at tidal volumes (Vt) > 8 mL kg^−1^. Nevertheless, most critically ill conditions demand lower Vt. We sought to evaluate the operative performance of several predictors of fluid responsiveness at Vt ≤ 8 mL kg^−1^ by using meta-regression and subgroup analyses.

**Methods:**

A sensitive search was conducted in the Embase and MEDLINE databases. We searched for studies prospectively assessing the operative performance of pulse pressure variation (PPV), stroke volume variation (SVV), end-expiratory occlusion test (EEOT), passive leg raising (PLR), inferior vena cava respiratory variability (Δ-IVC), mini-fluid challenge (m-FC), and tidal volume challenge (VtC), to predict fluid responsiveness in adult patients mechanically ventilated at Vt ≤ 8 ml kg^−1^, without respiratory effort and arrhythmias, published between 1999 and 2020. Operative performance was assessed using hierarchical and bivariate analyses, while subgroup analysis was used to evaluate variations in their operative performance and sources of heterogeneity. A sensitivity analysis based on the methodological quality of the studies included (QUADAS-2) was also performed.

**Results:**

A total of 33 studies involving 1,352 patients were included for analysis. Areas under the curve (AUC) values for predictors of fluid responsiveness were: for PPV = 0.82, Δ-IVC = 0.86, SVV = 0.90, m-FC = 0.84, PLR = 0.84, EEOT = 0.92, and VtC = 0.92. According to subgroup analyses, variations in methods to measure cardiac output and in turn, to classify patients as responders or non-responders significantly influence the performance of PPV and SVV (*p* < 0.05). Operative performance of PPV was also significantly affected by the compliance of the respiratory system (*p* = 0.05), while type of patient (*p* < 0.01) and thresholds used to determine responsiveness significantly affected the predictability of SVV (*p* = 0.05). Similarly, volume of fluids infused to determine variation in cardiac output, significantly affected the performance of SVV (*p* = 0.01) and PLR (*p* < 0.01). Sensitivity analysis showed no variations in operative performance of PPV (*p* = 0.39), SVV (*p* = 0.23) and EEOT (*p* = 0.15).

**Conclusion:**

Most predictors of fluid responsiveness reliably predict the response of cardiac output to volume expansion in adult patients mechanically ventilated at tidal volumes ≤ 8 ml kg^−1^. Nevertheless, technical and clinical variables might clearly influence on their operative performance

## Introduction

Fluid administration is one of the first-line therapy interventions used to reverse tissue hypoperfusion during acute circulatory failure. Nevertheless, fluid administration is not free of adverse effects, especially when fluids are excessively administered. Dynamic assessment of preload responsiveness appraising heart–lung interactions is commonly used during the resuscitation of mechanically ventilated patients with acute circulatory failure. In this scenario, assessment of fluid responsiveness might limit fluid administration, potentially reducing the risk of fluid overload, avoiding complications derived from tissue oedema and increasing mechanical ventilation-free days, among others [1].

Several predictors of fluid responsiveness have been described in the medical literature [2]. Dynamic indices evaluating the response of the cardio-circulatory system to reversible preload variations might be grouped based on the way in which preload variation is assessed [3]: (a) first, indices based on mechanical ventilation-induced variations of stroke volume and stroke volume-derived/related parameters, such as pulse pressure variation (PPV), stroke volume variation (SVV), tidal volume challenge (VtC); (b) second, indices based on mechanical ventilation-induced variations of non-stroke volume-derived parameters such as the inferior vena cava respiratory variability (Δ-IVC); (c) third, indices based on preload-redistributing manoeuvers different from standard mechanical ventilation such as passive leg raising (PLR), end-expiratory occlusion test (EEOT), and mini-fluid challenge (m-FC). Indices from the first and second groups are, in principle, limited by the use of low tidal volumes [4, 5], high respiratory rates [6], low pulmonary compliance [7], and low driving pressures [8]. Conversely, indices from the third group could theoretically have better operative performances in most situations commonly observed in critically ill patients [7].

Several meta-analyses evaluating the operative performance of fluid responsiveness predictors in different clinical settings have led to variable results [9–20]. These meta-analyses, however, did not evaluate specific subgroups, and there are no meta-regressions assessing the reliability of methods to evaluate fluid responsiveness. Consequently, we sought to conduct a meta-analysis in order to analyse the operative performance of dynamic predictors of fluid responsiveness in critically ill adults mechanically ventilated at Vt ≤ 8 ml kg^−1^ without arrhythmias and increased respiratory effort. Additionally, we aim to identify clinical variables or methods affecting the operative performance of dynamic predictors of fluid responsiveness under such particular conditions.

## Methodology

### Protocol

This systematic review and meta-analysis was conducted in accordance with the Preferred Reporting Items for Systematic Reviews and Meta-Analysis (PRISMA) guidelines [21] and was recorded at PROSPERO (registration number CRD42019138147 (https://www.crd.york.ac.uk/prospero/display_record.php?ID=CRD42019138147)) on August 12, 2020.

Study selection and inclusion criteria

Studies prospectively evaluating the operative performance of PPV, SVV, VtC PLR, EEOT, m-FC, and Δ-IVC as predictors of fluid responsiveness in critically ill ventilated patients at Vt ≤ 8 ml kg^1^ and without respiratory effort and arrhythmias were selected for full-text reading. In addition, studies including subgroups of patients fulfilling our inclusion criteria were also selected and included for the analysis. No language restriction was applied. Only studies recording data about the operative performance of any fluid responsiveness test and including an explicit definition of fluid responsiveness after fluid loading were finally incorporated for the analysis. Studies conducted in the operating room, case reports, and studies including patients < 18 years old, pregnant women were excluded.

Search strategy, data extraction and quality appraisal

A comprehensive search was conducted in the MEDLINE and Embase databases, between January 1999 and May December 2019. Moreover, reference lists of each initially selected manuscript were manually reviewed searching for potential studies not retrieved by the original search. The complete search strategy and the terms used are available in the protocol recorded at PROSPERO. Two reviewers (J.I.A.S. and J.D.C.R.) independently assessed search results for inclusion and undertook data extraction and quality appraisal.

### Data items

Data extracted from each clinical trial included: authors, year of publication, number of patients enrolled, type of critically ill patient, age, height; norepinephrine dose, dobutamine, epinephrine, and vasopressin doses; main diagnosis; APACHE (Acute Physiology And Chronic Health Evaluation) II score; SOFA (Sequential Organ Failure Assessment) score; method used to evaluate fluid responsiveness; amount and type of fluids used during the fluid challenge; diagnostic test or fluid responsiveness predictor assessed; definition of fluid responsiveness used; % of response (i.e. cardiac output, VTI, etc.); cut-off point or threshold used to determine fluid responsiveness; tidal volume (Vt); respiratory system compliance; positive end-expiratory pressure (PEEP) level; airway driving pressure; presence of acute respiratory distress syndrome (ARDS); and finally, the sensitivity and specificity, and the area under the ROC curve (AUC) of the diagnostic test used.

### Quality assessment

Two authors (JIAS and JDCR) independently assessed the quality of each study by using the QUADAS-2 tool (Quality Assessment of Diagnostic Accuracy Studies) [22]. Disagreements were planned to be solved by consensus between these authors, with the possibility to consult a third author if discrepancies were maintained.

### Statistical analysis

#### Analysis of individual studies

Data regarding sensitivity, specificity, and diagnostic odds ratio (DOR) were calculated by using a contingency table. In some trials, prediction of fluid responsiveness was assessed by using different ventilation parameters or different thresholds, which resulted in multiple data about operative performances; in such cases, all data regarding operative performances were included for analysis.

#### Analysis of summary measures

Fitted sensitivity, specificity, and AUC data were assessed through bivariate and hierarchical analyses. The summary of receiver operating characteristic (ROC) curves was assessed by using the method of Rutter and Gatsonis [23]. Operative performance quality was graduated according to Fisher et al. [24]. Heterogeneity among trials was assessed using the Cochran’s Q tests and its effect was quantified by calculating the inconsistency (I^2^). An *I*^2^ > 50% was considered significant [25].

#### Analysis of risk of bias across studies

Asymmetry was assessed by the Thompson and Sharp test. Nevertheless, this was not applicable for PLR, Δ-IVC, VtC, and m-FC because the low number of studies addressing these predictors impedes the application of such test. Publication bias was fitted using the trim-and-fill method.

#### Additional analysis

Subgroup and meta-regression analyses were performed for all the clinical and physiological variables potentially influencing the operative performance of fluid responsiveness predictors: tidal volume, PEEP, driving pressure, compliance of the respiratory system, type of patient, method used to calculate the index, threshold used to predict fluid response, volume of fluid finally administered. This analysis was also used to determine the source of heterogeneity among studies.

A sensitivity analysis was carried out by performing a meta-regression based on the methodological quality of included studies (QUADAS-2). The threshold effect was assessed using Spearman´s rank correlation coefficient and the Moses–Shapiro–Littenberg method. Data analysis was performed using R software, version 3.4.3, together with the mada and meta packages. Data are expressed as a value (95% confidence interval (CI)), and *p* < 0.05 was considered statistically significant.

## Results

**Fig. 1:**
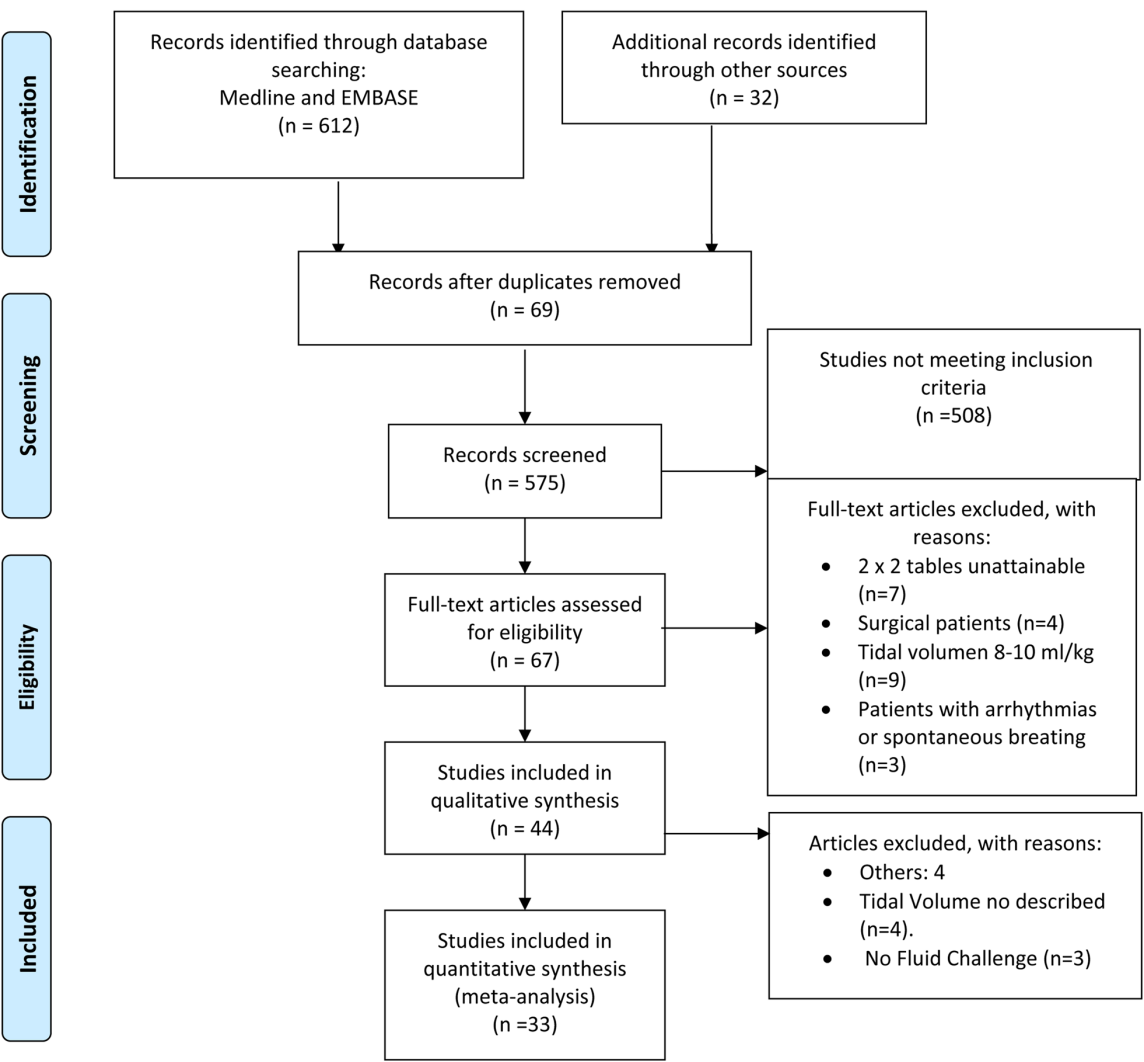
Study selection

A total of 644 studies were retrieved, including 612 from the MEDLINE and Embase databases, and 32 obtained from the reference lists of the studies retrieved from the original search. Finally, 33 studies fulfilling all the inclusion criteria were included for the quantitative analysis (Fig. 1).

### General characteristics of the studies included

A total of 33 studies involving 1352 patients were included for analysis. General characteristics of studies included are summarized in Tables 1 and 2. A total of 1413 fluid challenges were performed with an average fluid responsiveness of 53.06%.

**Table 1:** General characteristics of selected studies

#	Study	Year	Setting	Sample size	Predictor to fluid responsiveness	Method to measure cardiac output	Infusion volume	Tidal volume (ml/kg)	PEEP (mmHg)	Compliance (ml/cmH_2_O)	ARDS (%)
1	De Backer [4]	2005	Sepsis	33	PPV	TD (PAC)	500–100 ml LR and colloid	6.3 (6.0–7.1)	11.0	26.0	97.0
2	Auler et al.[26]	2008	Cardiovascular	59	PPV	TD (PAC)	20 ml/kg LR	8.0	5.0	NR	NR
3	Huang et al. [27]	2008	Sepsis	22	PPV	TD (PAC)	500 ml colloid	6.4 (0.7)	14.4	26.2	100.0
4	Vistisen et al. [28]	2009	Cardiovascular	23	PPV	C-TD (PAC)	500 ml colloid	6.9 (6.1–7.7) ABW	6.0	NR	NR
5	Vallée et al. [29]	2009	Sepsis	42	PPV	TPTD	6 ml/kg colloid	6.6 (6.2–7.3) IBW	6.0	27.0	47.00
6	Muller et al. [8]	2010	Sepsis	57	PPV	TD (PAC) and TPTD	250–500 ml saline and colloid	6.0 (4.8–7.8) IBW	4.0	26.4	NR
7	Moretti et al. [30]	2010	Neurology	29	ΔIVC	TPTD	7 ml/kg colloid	8.0	0.0	NR	NR
8	Lakhal et al. [31]	2011	Sepsis	65	PPV	TD (PAC) and TPTD	500 ml colloid	6.9 (5.9–7.8) PBW	8.5	40.4	100.0
9	Muller et al. [32]	2011	Sepsis	39	Mini-fluid challenge	VTI (TTE)	500 ml colloid	6.6 (6.3–7.1) IBW	5.0	12.0	100.0
10	Monnet et al.[7]	2012	Sepsis	28	PPV, PLR, EEOT	TPTD	500 ml saline	7.1 (6.3–7.9) PBW	7.0	23	89.0
11	Cecconi et al. [33]	2012	Postsurgical	31	PPV, SVV	C-PCA	250 ml colloid	8.0 PBW	5.0	NR	NR
12	Yazigi et al. [34]	2012	Cardiovascular	60	PPV	TD (PAC)	7 ml/kg colloid	8.0	0	NR	NR
13	Oliveira-Costa et al. [35]	2012	Sepsis	37	PPV	TD (PAC)	500–1000 ml LR and colloid	6.5 (6.0–6.5) IBW	7.5	34.0	27.0
14	Drvar et al. [36]	2013	Sepsis	46	PPV, SVV	C-PCA	500 ml colloid	7.0	5.0	NR	NR
15	Freitas et al. [37]	2013	Sepsis	40	PPV	TD (PAC)	7 ml/kg colloid	6.0 PWB	10.0	31.0	100.0
16	Trepte et al.[38]	2013	Postsurgical	72	PPV, SVV	TPTD	300 ml colloid	8.0	5.0	NR	NR
17	Guarracino et al. [39]	2014	Sepsis	50	PPV	PRAM	7 ml/kg colloid	7.0 (6.0–8.0)	6.0	NR	NR
18	Kang et al. [40]	2014	Cardiovascular	54	SVV	TD (PAC)	240 ml colloid	6.0 IBW	0.0. or 5.0 or 10.0	NR	NR
19	Ibarra-Estrada et al. [41]	2015	Sepsis	59	PPV, SVV, PLR	TPTD	7 ml/kg saline	6.0 (6.0–6.3) PWB	6.0	NR	73.0
20	Angappan et al. [42]	2015	Sepsis	45	SVV	NC -PCA	500 ml colloid	8.0	NR	NR	NR
21	Mallat et al. [43]	2015	Sepsis	49	Mini-fluid challenge	TPTD	500 ml colloid	6.8 (6.4–7.3) IBW	8.0	13.8	48.0
22	Vistisen et al. [44]	2017	Cardiovascular	41	PPV	C-TD	500 ml LR and colloid	6.7 (0.94)	NR	NR	NR
23	Liu et al. [45]	2016	Sepsis	96	PPV	TPTD	500 ml saline	7.0 (6.2–7.8) PBW	NR	28	100.0
24	Cherpanath et al. [46]	2016	Cardiovascular	22	PPV, SVV	NC -PCA	500 ml colloid	7.0 (6.0–8.0) ABW	5.0	NR	NR
25	Oliveira et al. [47]	2016	Postsurgical	20	PPV, ΔIVC	VTI (TTE)	500 ml LR	8.0	5.0	46.2	NR
26	Sobczyk et al. [48]	2016	Cardiovascular	35	PLR, ΔIVC	VTI (TTE)	250 ml saline	8.0	5.0	NR	NR
27	Myatra et al. [49]	2017	Sepsis	30	PPV, SVV, EEOT, tidal volume challenge	TPTD	7 ml/kg saline	8.0 IBW	8.0	29.0	10.0
28	Yonis et al. [50]	2017	Sepsis	19	PPV, tidal volume challenge	TPTD	500 ml LR	6.0 (5.9–6.1) PBW or 8.0 PBW	9.0	30.0	100.0
29	Jozwiak et al. [51]	2017	Sepsis	30	EEOT	TPTD	500 ml saline	6.3 (5.8–6.5) PBW	10.0	12.0	20.0
30	Guo-Guang Ma et al. [52]	2018	Cardiovascular	70	SVV, PLR, ΔIVC	NC -PCA	500 ml colloid	8.0	5.0	NR	NR
31	Georges et al. [53]	2018	Neurology	50	EEOT	VTI (TTE)	500 ml saline	6.9 (0.7) IBW	6.0	10.0	NR
32	Depret et at [54]	2019	Sepsis	28	EEOT	TPTD	500 ml saline	5.8 (0.7)	11.0	11.0	71.0
33	Fot et al. [55]	2019	Cardiovascular	32	Mini-fluid challenge	TPTD	7 ml/kg LR	7.6 (6.9–8.0) PBW	5.0	NR	NR

Values are expressed as pooled value (95% confidence interval). ABW, actual body weight; ARDS, acute respiratory distress syndrome; C-TD, continuous thermodilution; C-PCA, calibrated pulse contour analysis;; IBW, ideal body weight; PBW, predicted body weight; PEEP, positive end-expiratory pressure; EEOT, end expiratory occlusion test; NC-PCA, non-calibrated pulse contour analysis; NR, not reported; PPV, pulse pressure variation; SVV, stroke volume variation; LR, lactate ringer; PAC, pulmonary artery catheter; PLR, passive leg raising; PRAM, pressure recording analytical method; TD, thermodilution; TPTD, transpulmonary thermodilution; TTE, transthoracic echocardiography; VTI, velocity time integral; ΔIVC, inferior vena cava variability. Values are expressed as pooled data (95% confidence interval) or median (IQR)

**Table 2:** Operative performance of predictors of fluid responsiveness in mechanically ventilated patients at Vt ≤ 8 ml/kg without arrhythmia and respiratory effort

Study	year	Predictor of fluid responsiveness	tp	n1	tn	n2	nt	Sensibility	Specificity	AUC	Threshold (%)	Method used to measure the variable studied	Haemodynamic end point	Fluid responsiveness rate
De Backer et al. [4]	2005	PPV	12	18	10	15	33	0.67 (0.41–0.87)	0.67 (0.38–0.88)	0.71 ± 0.09	8	Analysis of arterial tracing	CI ≥ 15%	0.55
Auler et al. [26]	2008	PPV	38	39	19	20	59	0.97 (0.87–1.00)	0.95 (1.00)	0.98 ± 0.01	12	Analysis of arterial tracing	CI ≥ 15%	0.66
Huang et al. [27]	2008	PPV	7	10	12	12	22	0.70 (0.35–0.93)	1.00 (0.74–1.00)	0.76	11.8	Contour pulse wave analysis (PiCCO system)	CI ≥ 15%	0.45
Vistisen et al. [28]	2009	PPV	16	17	5	6	23	0.94 (0.71–1.00)	0.83 (0.36–1.00)	NR	6.5	Analysis of arterial tracing	CI ≥ 15%	0.73
Vallee et al. [29]	2009	PPV	5	16	19	26	42	0.31 (0.11–0.59)	0.73 (0.52–0.88)	0.62 (0.45–0.80)	15	Analysis of arterial tracing	CI ≥ 15%	0.38
Muller et al. [8]	2010	PPV	25	41	15	16	57	0.61 (0.45–0.76)	0.94 (0.70–1.00)	0.77 (0.65–0.90)	7	Analysis of arterial tracing	CI or ISV ≥ 15%	0.72
Lakhal et al. [31]	2011	PPV	19	26	33	39	65	0.73 (0.52–0.88)	0.85 (0.69–0.94)	0.75 (0.62–0.85)	5	Analysis of arterial tracing	CO ≥ 10%	0.4
Monnet et al. [7]	2012	PPV	15	15	4	13	28	1.00 (0.78–1.00)	0.31 (0.09–0.61)	0.69 (± 0.10)	4	Contour pulse wave analysis (PiCCO system)	CI ≥ 15%	0.53
Cecconi et al. [33]	2012	PPV	17	20	8	11	31	0.85 (0.62–0.97)	0.73 (0.39–0.94)	0.87 (0.76–0.99)	13	Contour pulse wave analysis (LiDCO system)	SV ≥ 15%	0.39
Yazigi et al. [34]	2012	PPV	33	41	14	19	60	0.80 (0.65–0.91)	0.74 (0.49–0.91)	0.85 (0.75–0.94)	11.5	Analysis of arterial tracing	ISV ≥ 15%	0.68
Oliveira-Costa et al. [35]	2012	PPV	9	17	19	20	37	0.53 (0.28–0.77)	0.95 (0.75–1.00)	0.74 (0.56–0.90)	10	Analysis of arterial tracing	CI ≥ 15%	0.45
Drvar et al. [36]	2013	PPV	26	26	20	20	46	1.00 (0.87–1.00)	1.00 (0.83–1.00)	1.00 (0.92–1.00)	12	Contour pulse wave analysis (LiDCO system)	ISV ≥ 15%	0.57
Freitas et al. [37]	2013	PPV	17	19	10	11	40	0.89 (0.67–0.99)	0.91 (0.59–1.00)	0.91 (0.82–1.00)	6.5	Contour pulse wave analysis (computer software)	CO ≥ 15%	0.47
Trepte et al. [38]	2013	PPV	25	41	25	31	72	0.61 (0.45–0.76)	0.81 (0.63–0.93)	0.70 (0.21–0.85)	10.1	Contour pulse wave analysis (PiCCO system)	CI ≥ 10%	0.57
Guarracino et al. [39]	2014	PPV	29	30	11	20	50	0.97 (0.83–1.00)	0.55 (0.32–0.77)	0.85 (0.72–0.93)	12.5	Contour pulse wave analysis (computer software)	CI ≥ 15%	0.6
Ibarra-Estrada et al. [41]	2015	PPV	15	30	15	19	59	0.50 (0.31–0.69)	0.79 (0.54–0.94)	0.63 (0.49–0.75)	14	Analysis of arterial tracing	ISV ≥ 15%	0.51
Vistisen et al. [44]	2016	PPV	11	17	18	24	41	0.65 (0.38–0.86)	0.75 (0.53–0.90)	0.57 (0.39–0.75)	12	Analysis of arterial tracing	SV ≥ 15%	0.41
Liu et al. [45]	2016	PPV	35	52	37	44	96	0.67 (0.53–0.80)	0.84 (0.70–0.93)	0.78 (0.69–0.86)	10	Contour pulse wave analysis (PiCCO system)	CO ≥ 15%	0.54
Cherpanath el al [46]	2016	PPV	18	19	3	3	22	0.95 (0.74–1.00)	1.00 (0.29–1.00)	0.98 (0.82–1.00)	8	Contour pulse wave analysis (computer software)	CO ≥ 15%	0.86
Oliveira et al. [47]	2016	PPV	8	9	11	11	20	0.89 (0.52–1.00)	1.00 (0.72–1.00)	0.92	12.4	Contour pulse wave analysis (computer software)	VTI ≥ 15%	0.45
Myatra et al. [49]	2017	PPV	12	16	14	14	30	0.75 (0.48–0.93)	1.00 (0.77–1.00)	0.91 (0.81–1.00)	11.5	Contour pulse wave analysis (PiCCO system)	CI ≥ 15%	0.53
Yonis et al. [50]	2017	PPV	3	9	8	10	19	0.33 (0.07–0.70)	0.80 (0.44–0.97)	0.49 (0.21–0.77)	10	Contour pulse wave analysis (PiCCO system)	CI ≥ 15%	47.36
Yonis et al. [50]	2017	PPV	7	9	4	10	19	0.78–0.97)	0.40 (0.12–0.74)	0.52 (0.24–0.80)	10	Contour pulse wave analysis (PiCCO system)	CI ≥ 15%	47.36
Cecconi et al. [33]	2012	SVV	15	20	9	11	31	0.75 (0.51–0.91)	0.82 (0.48–0.98)	0.84 (0.71–0.96)	12.5	Contour pulse wave analysis (LiDCO system)	SV ≥ 15%	39.00
Trepte et al. [38]	2013	SVV	26	41	23	31	72	0.63 (0.47–0.78)	0.74 (0.55–0.88)	0.72 (0.21–0.85)	9.9	Contour pulse wave analysis (PiCCO system)	CI ≥ 10%	57.00
Drvar et al. [36]	2013	SVV	25	26	20	20	46	0.96 (0.80–1.00)	1.00 (0.83–1.00)	0.96 (0.85–0.99)	10	Contour pulse wave analysis (LiDCO system)	ISV ≥ 15%	57.00
Kang et al. [40]	2014	SVV	24	27	24	27	54	0.89 (0.71–0.98)	0.89 (0.71–0.98)	0.90 (0.80–0.99)	13.5	Bioreactance (NICOM system)	CO ≥ 7%	50.00
Kang et al. [40]	2014	SVV	25	27	23	27	54	0.93 (0.76–0.99)	0.85 (0.66–0.96)	0.93 (0.83–1.00)	13.5	Bioreactance (NICOM system)	CO ≥ 7%	50.00
Kang et al. [40]	2014	SVV	25	27	25	27	54	0.93 (0.76–0.99)	0.93 (0.76–0.99)	0.94 (0.86–1.00)	13.5	Bioreactance (NICOM system)	CO ≥ 7%	50.00
Ibarra-Estrada et al. [41]	2015	SVV	23	30	13	19	59	0.77 (0.58–0.90)	0.68 (0.43–0.87)	0.72 (0.59–0.83)	16	Contour pulse wave analysis (PiCCO system)	ISV ≥ 15%	51.00
Angappan et al. [42]	2015	SVV	23	29	14	16	45	0.79 (0.60–0.92)	0.88 (0.62–0.98)	0.71 (0.56–0.84)	13	Contour pulse wave analysis (Vigileo)	CI ≥ 15%	64.00
Cherpanath et al. [46]	2016	SVV	17	19	3	3	22	0.89 (0.67–0.99)	1.00 (0.29–1.00)	0.95 (0.76–0.99)	9	Contour pulse wave analysis (computer software)	CO ≥ 15%	86.00
Myatra et al. [49]	2017	SVV	12	16	13	14	30	0.75 (0.48–0.93)	0.93 (0.66–1.00)	0.92 (0.82–1.00)	10.5	Contour pulse wave analysis (PiCCO system)	CI ≥ 15%	53.00
Guo-Guang Ma et al. [52]	2018	SVV	32	35	33	35	70	0.91 (0.77–0.98)	0.94 (0.81–0.99)	0.97 (0.89–0.99)	12	Contour pulse wave analysis (Vigileo)	SV ≥ 15%	50.00
Monnet et al. [7]	2012	PLR	14	15	13	13	28	0.93 (0.68–1.00)	1.00 (0.75–1.00)	0.94 (± 0.05)	CI ≥ 10	Contour pulse wave analysis (PiCCO system)	CI ≥ 15%	53.00
Ibarra-Estrada et al. [41]	2015	PLR	19	30	14	19	59	0.63 (0.44–0.80)	0.74 (0.49–0.91)	0.69 (0.56–0.80)	ISV ≥ 15	Contour pulse wave analysis (PiCCO system)	ISV ≥ 15%	51.00
Sobczyk et al. [48]	2016	PLR	19	24	9	11	35	0.79 (0.58–0.93)	0.82 (0.48–0.98)	0.80	CO ≥ 15	CO (echocardiogram)	CO ≥ 15%	68.57
Guo-Guang Ma et al. [52]	2018	PLR	35	35	29	35	70	1.00 (0.90–1.00)	0.83 (0.66–0.93)	0.91 (0.82–0.97)	SV ≥ 12.84	Contour pulse wave analysis (Vigileo)	SV ≥ 15%	50.00
Monnet et al. [7]	2012	EEOT	14	15	12	13	28	0.93 (0.68–1.00)	0.92 (0.64–1.00)	0.93 (± 0.05)	CI ≥ 5	Contour pulse wave analysis (PiCCO system)	CI ≥ 15%	53.00
Myatra et al. [49]	2017	EEOT	14	16	13	14	30	0.88 (0.62–0.98)	0.93 (0.66–1.00)	0.95 (0.88–1.00)	Ci ≥ 4.1	Contour pulse wave analysis (PiCCO system)	CI ≥ 15%	53.00
Jozwiak et al. [51]	2017	EEOT	14	15	15	15	30	0.93 (0.68–1.00)	1.00 (0.78–1.00)	0.98 (0.85–1.00)	CI ≥ 4	Contour pulse wave analysis (PiCCO system)	CI ≥ 15%	50.00
Jozwiak et al. [51]	2017	EEOT	14	15	15	15	30	0.93 (0.68–1.00)	1.00 (0.78–1.00)	0.93 (0.78–0.99)	VTI ≥ 5	VTI (echocardiogram)	CI ≥ 15%	50.00
Georges et al. [53]	2018	EEOT	25	28	21	22	50	0.89 (0.72–0.98)	0.95 (0.77–1.00)	0.96 (± 0.03)	VTI ≥ 9	VTI (echocardiogram)	CO ≥ 15%	56.00
Georges et al. [53]	2018	EEOT	18	28	17	22	50	0.64 (0.44–0.81)	0.77 (0.55–0.92)	0.70 (± 0.07)	V_Max_ ≥ 8.5	V_Max_ (echocardiogram)	CO ≥ 15%	56.00
Depret et al. [54]	2019	EEOT	12	14	13	14	28	0.86 (0.57–0.98)	0.93 (0.66–1.00)	0.95 (0.79–0.99)	CI ≥ 3	Contour pulse wave analysis (PiCCO system)	CI ≥ 15%	50.00
Depret et al. [54]	2019	EEOT	14	14	14	14	28	1.00 (0.77–1.00)	1.00 (0.77–1.00)	1.00 (0.88–1.00)	CI ≥ 4	CI (esophageal Doppler)	CI ≥ 15%	50.00
Depret et al. [54]	2019	EEOT	10	14	12	14	28	0.71 (0.42–0.92)	0.86 (0.57–0.98)	0.80 (0.61–0.93)	FTC ≥ 3	FTC (esophageal Doppler)	CI ≥ 15%	50.00
Depret et al. [54]	2019	EEOT	10	14	12	14	28	0.71 (0.42–0.92)	0.86 (0.57–0.98)	0.75 (0.55–0.89)	V_Max_ ≥ 2	V_Max_ (esophageal Doppler)	CI ≥ 15%	50.00
Muller et al. [32]	2011	Mini-fluid challenge	20	21	14	18	39	0.95 (0.76–1.00)	0.78 (0.52–0.94)	0.92 (0.78–0.98)	VTI ≥ 3	VTI (echocardiogram)	VTI ≥ 15%	54.00
Mallat et al. [43]	2015	Mini-fluid challenge	19	22	24	27	49	0.86 (0.65–0.97)	0.89 (0.71–0.98)	0.91 (0.81–0.98)	Changes in SVV of -2	Contour pulse wave analysis (PiCCO system)	CI ≥ 15%	45.00
Mallat et al. [43]	2015	Mini-fluid challenge	19	22	23	27	49	0.86 (0.65–0.97)	0.85 (0.66–0.96)	0.92 (0.81–0.98)	Changes in PPV of -3	Contour pulse wave analysis (PiCCO system)	CI ≥ 15%	45.00
Mallat et al. [43]	2015	Mini-fluid challenge	17	22	20	27	49	0.77 (0.55–0.92)	0.74 (0.54–0.89)	0.78 (0.64–0.88)	CI ≥ 5.2	Contour pulse wave analysis (PiCCO system)	CI ≥ 15%	45.00
Fot et al. [55]	2019	Mini-fluid challenge	12	14	12	18	32	0.86 (0.57–0.98)	0.67 (0.41–0.87)	0.77	Changes in PPV of -2	Contour pulse wave analysis (PiCCO system)	CI ≥ 15%	43.00
Fot el al [55]	2019	Mini-fluid challenge	11	14	12	18	32	0.79 (0.49–0.95)	0.67 (0.41–0.87)	0.75	Changes in SVV of -2	Contour pulse wave analysis (PiCCO system)	CI ≥ 15%	43.00
Myatra et al. [49]	2017	Tidal volume challenge	15	16	14	14	30	0.94 (0.70–1.00)	1.00 (0.77–1.00)	0.99 (0.98–1.00)	Changes in PPV of 3.5	Contour pulse wave analysis (PiCCO system)	CI ≥ 15%	53.00
Myatra et al. [49]	2017	Tidal volume challenge	14	16	14	14	30	0.88 (0.62–0.98)	1.00 (0.77–1.00)	0.97 (0.92–1.00)	Changes in SVV of 2.5	Contour pulse wave analysis (PiCCO system)	CI ≥ 15%	53.00
Yonis et al. [50]	2017	Tidal volume challenge	9	9	4	10	19	1.00 (0.66–1.00)	0.40 (0.12–0.74)	0.59 (0.31–0.88)	Changes in PPV of 29	Contour pulse wave analysis (PiCCO system)	CI ≥ 15%	47.36
Moretti et al. [30]	2010	ΔIVC	12	17	12	12	29	0.71 (0.44–0.90)	1.00 (0.74–1.00)	0.90	16	Ultrasonography	CI ≥ 15%	58.00
De Oliveira et al. [47]	2016	ΔIVC	6	9	11	11	20	0.67 (0.30–0.93)	1.00 (0.72–1.00)	0.84 (± 0.10)	16	Ultrasonography	VTI ≥ 15%	45.00
Sobczyk et al. [48]	2016	ΔIVC	20	24	8	11	35	0.83 (0.63–0.95)	0.73 (0.39–0.94)	0.73	18	Ultrasonography	CO ≥ 15%	68.57
Guo-Guang et al. [52]	2018	ΔIVC	30	35	30	35	70	0.86 (0.70–0.95)	0.86 (0.70–0.95)	0.83 (0.72–0.91)	13.39	Ultrasonography	SV ≥ 15%	50.00

Values are expressed as pooled value (95% confidence interval). CI, cardiac index; CO, cardiac output; PAC, pulmonary artery catheter; PiCCO; pulse contour cardiac output; EEOT, end expiratory occlusion; FTC, flow time corrected; ISV, index stroke volume; n1, number of patient with positive fluid responsiveness; n2, number of patients with negative fluid responsiveness; NR, not reported; PLR, passive leg raising; PPV, pression pulse variation; SV, stroke volume; SVV, stroke volume variability; tn, true negative; tp, true positive; VTI, velocity time integral; V_Max_, peak velocity; ΔIVC, inferior vena cava variability. Values are expressed as pooled data (95% confidence interval) or median (IQR)

### Risk of bias

The risk of bias of the included studies is summarized in Additional file 1: Table S1.

### Syntheses of results

**Fig. 2:**
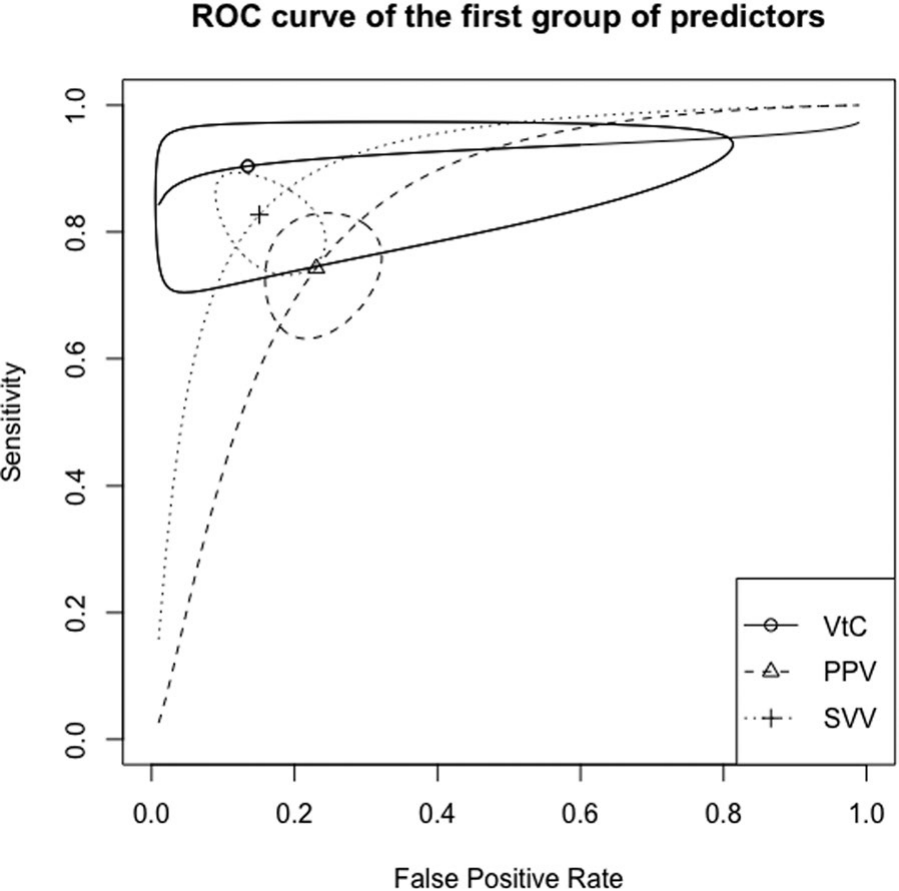
Summary ROC curve for the first group of predictors of fluid responsiveness. SVV, stroke volume variation; PPV, pulse pressure variation; VtC, tidal volume challenge. Closed curve: 95% confidence region

**Fig. 3:**
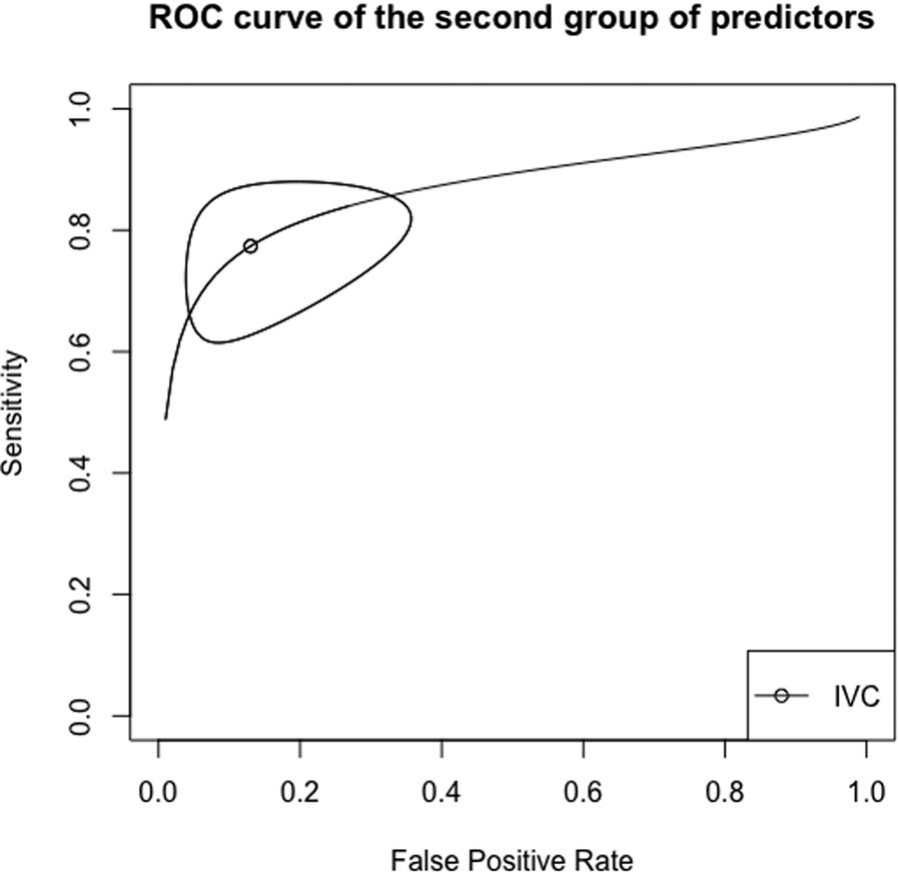
Summary ROC curve for the second group of predictors of fluid responsiveness. IVC, inferior vena cava respiratory variability. Closed curve: 95% confidence region

**Fig. 4:**
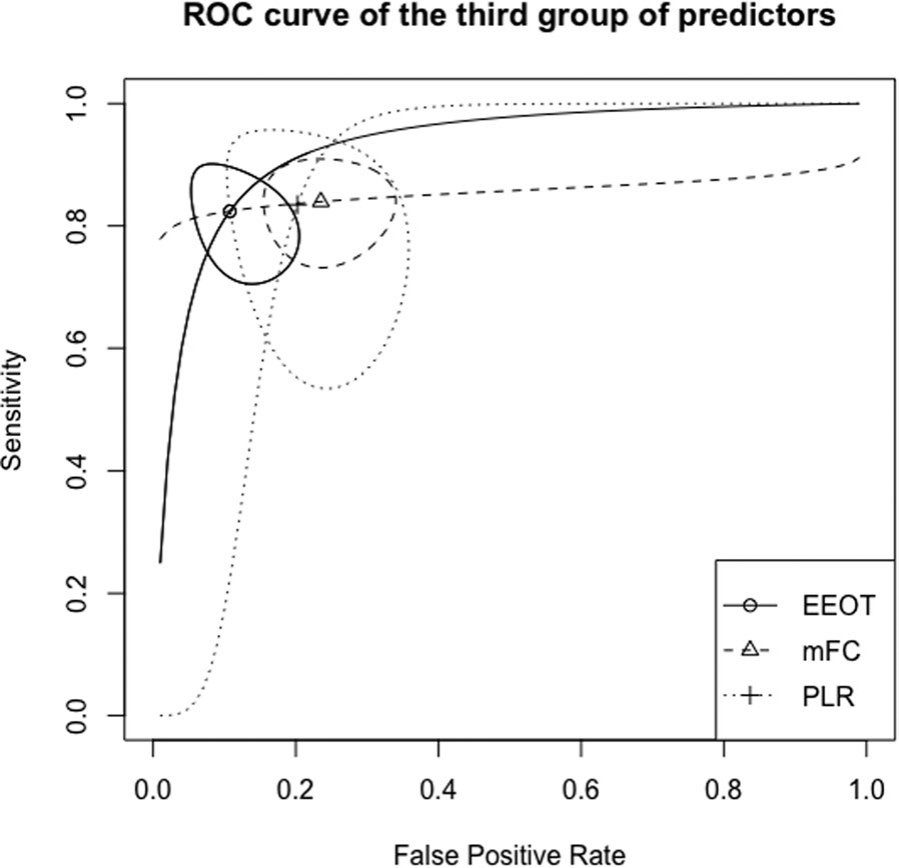
Summary ROC curve for the third group of predictors of fluid responsiveness. EEOT, end-expiratory occlusion test; m-FC, mini-fluid challenge; PLR, passive leg raising. Closed curve: 95% confidence region

Operative performance of fluid responsiveness predictors is shown in Table 3. Receiving operator (ROC) curves for the three groups of predictors are presented in Figs. 2, 3 and 4. Moderate heterogeneity was found among studies assessing PPV (see Additional file 2: Figure S1), SVV (Additional file 3: Figure S2), PLR (Additional file 4: Figure S3, and EEOT (Additional file 5: Figure S4). Conversely, heterogeneity was not found among studies that assessed the other predictors (see Additional file 6: Figures S5, Additional file 7: Figure S6 and Additional file 8: Figure S7).

**Table 3:** Operative performance of predictors of fluid responsiveness

Predictor of fluid responsiveness	Sensibility	Specificity	AUC	Threshold (%)	DOR	I^2^ (%)
First group						
PPV	0.74 (0.66–0.81)	0.77 (0.70–0.83)	0.82	10	11.70 (6.73–20.37)	56
Tidal volume challenge	0.90 (0.76–0.97)	0.87 (0.31–0.99)	0.92	3	82.95 (12.37–556.12)	8
SVV	0.83 (0.75–0.88)	0.85 (0.78–0.90)	0.90	12	28.82 (12.43–66.84)	63
Second group						
ΔIVC	0.77 (0.65–0.86)	0.87 (0.70–0.95)	0.86	16	24.13 (9.71–59.67)	0
Third group						
Mini-fluid challenge	0.84 (0.76–0.90)	0.76 (0.68–0.83)	0.84	1	15.57 (8.02–30.25)	9
PLR	0.83 (0.61–0.94)	0.80 (0.68–0.88)	0.84	13	31.65 (4.16–240.93)	74
EEOT	0.82 (0.73–0.89)	0.89 (0.82–0.94)	0.92	5	39.35 (14.80–104.60)	51

Values are expressed as pooled value (95% confidence interval). AUC, area under curve; I^2^, inconsistency; DOR, diagnostic odds ratio; EEOT, end expiratory occlusion; PLR, passive leg raising; PPV, pulse pressure variation; SVV, stroke volume variability. Values are expressed as pooled data (95% confidence interval)

### Risk of bias across studies

Asymmetry was present among studies assessing PPV (*p* = 0.02), SVV (*p* = 0.04), and EEOT (*p* < 0.03), and it was caused by publication bias (see Additional file 9: Figures S8, Additional file 10: Figure S9 and Additional file 11: Figure S10). Meanwhile, asymmetry was not performed for other predictors due to the low number of studies evaluating them.

Asymmetry among studies on PPV was fitted by using the trim-and-fill method, improving heterogeneity (*I*^2^ = 37.3%; *p* = 0.02), and the DOR obtained using the random effects model was decreased (DOR = 6.68; 95% CI 3.85–11.58). On the other hand, when the asymmetry of studies that assessed SVV was fitted, DOR by random effects also decreased (DOR = 11.3; 95% CI 4.34–29.66), but there were no changes in the heterogeneity (I^2^ = 73.1%; *p* < 0.001). Finally, when asymmetry among studies that assessed EEOT was fitted, both DOR by random effects (DOR = 12.93; 95% CI 5.31–31.50) and heterogeneity decreased (*I*^2^ = 29%; *p* = 0.13).

### Additional analysis

**Fig. 5:**
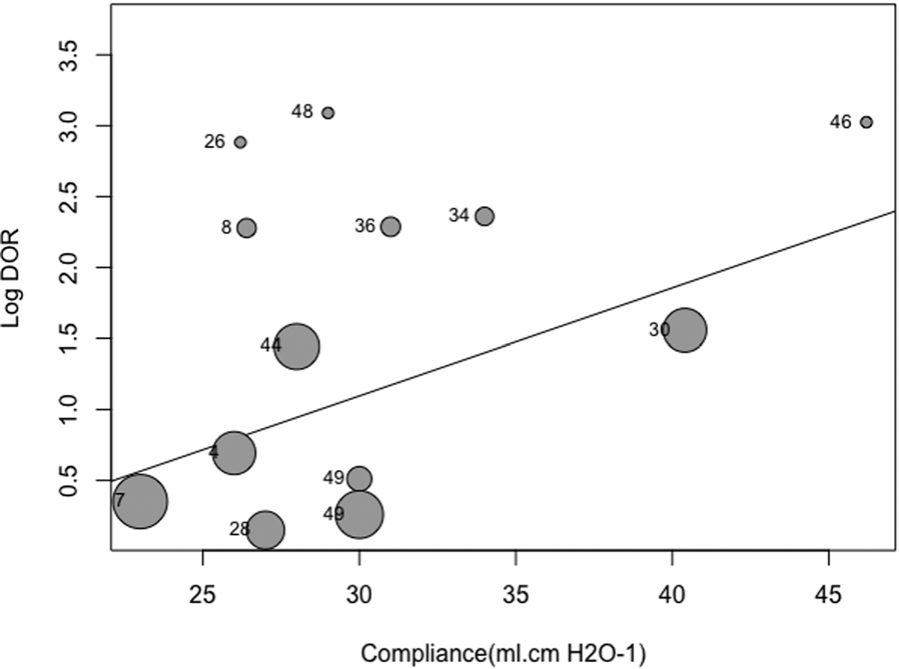
Bubble plot for meta-regression of pulse pressure variation with lung compliance pulmonary as a continuous covariate. The number of the point is the reference number of each study

Subgroup and meta-regression analyses attaining statistical significance are shown in Table 4. Operative performance of PPV was affected by the method used to calculate cardiac output (*p* = 0.02) and by the compliance of the respiratory system (*p* = 0.05) (Fig. 5). Additionally, these variables were a source of heterogeneity (*p* < 0.05).

**Table 4:** Subgroups and meta-regression analysis

Subgroup	Predictor of intravenous fluid	Number of studies	Odds ratio (95% CI)	P value by meta-regression	P value by subgroup analysis	I^2^ (%)	Q (value p)
Method to measure cardiac output							
TD	PPV	6	22.64 (7.86–65.25)	0.001	0.02	43.86	28.50. * p* = 0.03
TD and TDTP		2	17.58 (3.60–85.83)	0.79			
TPTD		8	4.96 (2.20–11.17)	0.03			
C-PCA		1	2173 (30.73–153,655.35)	0.04			
TTE		1	130.33 (3.32–5114.78)	0.37			
C-TD		2	11.57 (1.99–67.14)	0.52			
Others		3	28.38 (6.67–141.94)	0.82			
Others	SVV	2	18.45 (3.73–91.24)	0.12	< 0.01	0.0	5.57. *p* = 0.47
TPTD		3	7.02 (3.29–14.97)	< 0.01			
C-PCA		1	697.00 (26.95–18,029)	0.23			
TD		3	84.61 (29.50–242.72)	< 0.01			
NC -PCA		2	64.49 (18.18–228.71)	0.75			
Compliance	PPV	13	DOR = 1.08 (IC 95% 1.00–1.16)	0.05	NA	47.90	21.11. * p* = 0.03
Threshold used							
> 7%	SVV	3	86.54 (21.58–347.11)	0.02	0.05	39.17	13.15. * p* = 0.11
> 10%		1	4.98 (0.77–32.15)	0.09			
> 15%		7	33.10 (12.50–87-67)	0.08			
Critical care setting							
Sepsis	SVV	4	21.23 (7.66–58.81)	< 0.01	< 0.01	15.33	9.45. * p* = 0.31
Postsurgical		2	6.70 (2.25–19.98)	0.13			
Cardiovascular		5	95.67 (36.77–250.54)	0.03			
Volume of fluid load							
250 ml	SVV	4	54.10 (18.76–156.00)	< 0.01	0.01	21.79	8.95. * p* = 0.26
300 ml		1	4.98 (1.14–21.82)	0.01			
500 ml		4	86.73 (24.66–305.11)	0.57			
7 ml/kg		2	11.82 (2.92–47.80)	0.09			
250 ml	PLR	1	17.10 (2.77–105.70)	< 0.01	< 0.01	0.0	0.01. * p* = 0.93
500 ml		2	293.64 (33.14–2601.57)	0.05			
7 ml/kg		1	4.84 (1.37–17.09)	0.26			

C-PCA; calibrated pulse contour analysis; C-TD, continuous thermodilution; NC-PCA, non-calibrated pulse contour analysis; I^2^, inconsistency; PAC, pulmonary artery catheter; PPV, pulse pressure variation; PLR, passive leg raising; Q, Cochrane statistics; TD; thermodilution; TPTD, transpulmonary thermodilution; TTE; TTE, transthoracic echocardiography: SVV, stroke volume variability. Values are expressed as pooled data (95% confidence interval)

Operative performance of SVV was affected by the method to calculate cardiac output (*p* = 0.01), the threshold selected to define positive fluid responsiveness (*p* = 0.05), the type of critically ill patient (*p* < 0.001), and the volume of fluid finally used during the fluid challenge (*p* = 0.01). These subgroups were a source of heterogeneity since they disappeared among studies (I^2^ < 25%, *p* > 0.05).

Additionally, subgroup analysis of studies assessing PLR showed that volume of fluids infused to determine variation in cardiac output, significantly affected its operative performance (*p* < 0.01), and it was a source of heterogeneity since it disappeared among studies (*p* = 0.93). Subgroup and meta-regression analyses of the remaining predictors did not show any change in their operative performance or heterogeneity (Additional file 12: Table S2).

According to the sensitivity analysis based on the methodological quality of the included studies (QUADAS-2), there were no changes in the operative performance of PPV (*p* = 0.39), SVV (*p* = 0.23) and EEOT (*p* = 0.15) (see Additional file 12: Table S2). It should be noted that this analysis was not performed for other predictors due to the low number of studies evaluating them. According to the rho correlation coefficient or the Moses–Shapiro–Littenberg test, there was no threshold effect for any of the predictors (*p* > 0.05).

## Discussion

This systematic review and meta-analysis reveal that VtC, EEOT, and SVV have excellent operative performance, while ∆-IVC, PLR, m-FC, and PPV had good operative performance as predictors of fluid responsiveness in critically ill ventilated patients at Vt ≤ 8 ml kg^−1^ and without respiratory effort and arrhythmias. Methods to calculate cardiac output was important sources of heterogeneity. In addition, as expected, compliance of the respiratory system and type of patient affected the performance of SVV, while the volume of fluids infused to determine variation in cardiac output, significantly affected the performance of SVV and PLR.

Several meta-analyses have evaluated the operative performance of these predictors in different clinical settings [9–20]. Differently from this current metanalysis, patients included received Vt from 4.9 to 12 ml kg^−1^ [9, 10, 17] and evaluated other types of populations [14, 18]. Even though, our data suggest that most of fluid responsiveness predictors have good reliability even in conditions in which such prediction could be assumed that it would not be good.

The VtC and EEOT performances for determining fluid responsiveness were superior. Some studies showed that operative performance of EEOT was not good at Vt < 6 ml kg^−1^ [49, 56]. Meanwhile, a recent meta-analysis reported an adequate reliability of EEOT in mechanically ventilated patients at Vt ≤ 7 ml kg^−1^ [57], a finding in agreement with our results. Therefore, EEOT could be used for patients ventilated at any Vt. SVV depicted a better performance than PPV, which may be explained by the fact that PPV depends on effective arterial elastance [58], a variable that summarizes the features of arterial vascular load in humans [59]. We assessed studies that included critically ill patients who could have a low arterial load. Therefore, PPV susceptibility to haemodynamic changes may be increased when a low Vt is used.

Prediction of fluid responsiveness of some indices rely on tidal volume and intrathoracic pressure variations [4, 5]. Interestingly, operative performance of predictors analysed in this current metanalysis were apparently not affected by PEEP levels or driving pressures, which differ from other studies [8, 60] (see Additional file 12: Table S2). Nevertheless, respiratory system compliance directly affected the reliability of PPV (*p* = 0.05) to predict fluid responsiveness, which suggests that effects of respiratory pressure and tidal volume mainly rely on the degree to which these variables are transmitted to the pulmonary circulation and not on their absolute values [7].

Methods used to classify patients as fluid responders or not responders after the final fluid loading significantly affecting the reliability of PPV and SVV to predict fluid responsiveness. In this regard, operative performance was lower when transpulmonary thermodilution was used (through a PiCCO monitoring system) than when using the conventional thermodilution (through a pulmonary artery catheter) (see Table 4). Thus, more than errors implicit to the cardiac output calculations, classification as responder or non-responder derived from the method to estimate cardiac output was apparently a determinant of the reliability of such predictors. In addition, use of different thresholds to classify patients as fluid responders also influence on their operative performance (*p* = 0.05).

As expected, lower thresholds might increase operative performances in some cases (see Table 4). Importantly, reliability of SVV also varied depending on the type of critically ill patient (*p* < 0.01): better performance was found in post-cardiovascular surgery patients and in those with septic shock (DOR = 95.67; *p* = 0.03, and DOR = 21.23; *p* < 0.01, respectively), than in post high-risk surgery patients (DOR = 6.70; *p* = 0.13). We hypothesized that this finding represents a higher proportion of abdominal hypertension cases in the last group of patients since this might be a common complication in the postoperative period [61]. The presence of intraabdominal hypertension decreases thoracic compliance, resulting in increased SVV values regardless of preload dependency [62] and reduced operative performance. Finally, volume of a fluid loading with which fluid responsiveness was finally determined, significantly influenced the reliability of SVV and PLR. Nevertheless, these findings should be taken with caution, and we think that they should be considered as a source of heterogeneity.

An important point to retain is that positive fluid responsiveness should not systematically lead to fluid administration. Indeed, only during circulatory failure accompanied by altered tissue perfusion status, fluid administration should be considered aiming to increase cardiac output assuming this will revert tissue hypoperfusion and will restore normal cell respiration. Benefit of increasing cardiac output by volume expansion in positive fluid responders should be always balanced with the risk of fluid overload, which may be harmful.

This meta-analysis had several limitations. First, only adult critically ill ventilated patients with a Vt ≤ 8 ml kg^−1^ and without respiratory effort and arrhythmias were included, so the findings reported cannot be extrapolated to other clinical settings. Second, some predictors of fluid responsiveness were evaluated by a small number of studies, which limit their analysis. Third, the GRADE system (Grading of Recommendations, Assessment, Development, and Evaluations) was not used to determine or assess the meta-analysis’s quality since it was not established in our protocol. Conversely, we performed a sensitivity analysis based on the methodological quality of the included studies (QUADAS-2).

Fourth, moderate heterogeneity was found for some predictors, so these findings should be interpreted with caution. Nevertheless, other sources conversely decreased heterogeneity, which would allow extrapolation of our findings to clinical practice. Finally, operative performance of fluid responsiveness test was classified according to ROC curve analysis, which does not consider the DOR, a variable that summarizes the relation between sensitivity and specificity; however, in our opinion, DOR should always be considered for measuring operative performance when choosing a predictor of fluid responsiveness.

In conclusion, VtC, EEOT, and SVV have excellent operative performance, while ∆-IVC, PLR, m-FC, and PPV had good operative performance as predictors of fluid responsiveness in our setting. Method to calculate the cardiac output, threshold used to determine fluid responsiveness, volume administered during the fluid loading, and type of patient in which the test has been applied should have in account at moment to use it in clinical practice.

## Supplementary Information

**Additional file 1: Table S1.** Additional. Risk of bias of the trials as assessed by QUADAS-2 criteria.

**Additional file 2: Figure S1.** Diagnostic odds ratios of pulse pressure variation in adult critically ill ventilated patients with a Vt 8 ml kg^−1^ and without arrhythmia or respiratory effort.

**Additional file 3: Figure S2.** Diagnostic odds ratios of stroke volume variations in adult critically ill ventilated patients with a Vt 8 ml kg^−1^ and without arrhythmia or respiratory effort.

**Additional file 4: Figure S3.** Diagnostic odds ratios of passive leg raising in adult critically ill ventilated patients with a Vt 8 ml kg^−1^ and without arrhythmia or respiratory effort.

**Additional file 5: Figure S4.** Diagnostic odds ratios of End-expiratory occlusion test in adult critically ill ventilated patients with a Vt 8 ml kg^−1^ and without arrhythmia or respiratory effort.

**Additional file 6: Figure S5.** Diagnostic odds ratios of mini-fluid challenge in adult critically ill ventilated patients with a Vt 8 ml kg^−1^ and without arrhythmia or respiratory effort.

**Additional file 7: Figure S6.** Diagnostic odds ratios of tidal volume challenge in adult critically ill ventilated patients with a Vt 8 ml kg^−1^ and without arrhythmia or respiratory effort.

**Additional file 8: Figure S7.** Diagnostic odds ratios of inferior vena cava respiratory variability in adult critically ill ventilated patients with a Vt 8 ml kg^−1^ and without arrhythmia or respiratory effort.

**Additional file 9: Figure S8.** Contour enhanced funnel plot for a meta-analysis of pulse pressure variation for prediction of fluid responsiveness in patients with tidal volume 8 mL kg^−1^. Filled circles show an estimated treatment effect (Log diagnostic odds ratio) and its precision (standard error). In addition to individual study results, the fixed-effect estimates (vertical dashed line) with 95% confidence interval limits (diagonal dashed lines) and the random-effects estimate (vertical dotted line) are shown in the figure. The number of the point is the reference number of each study.

**Additional file 10: Figure S9.** Contour enhanced funnel plot for a meta-analysis of stroke volume variation for prediction of fluid responsiveness in patients with tidal volume 8 mL kg^−1^. Filled circles show an estimated treatment effect (Log diagnostic odds ratio) and its precision (standard error). In addition to individual study results, the fixed-effect estimates (vertical dashed line) with 95% confidence interval limits (diagonal dashed lines) and the random-effects estimate (vertical dotted line) are shown in the figure. The number of the point is the reference number of each study.

**Additional file 11: Figure S10.** Contour enhanced funnel plot for a meta-analysis of end-expiratory occlusion test for prediction of fluid responsiveness in patients with tidal volume 8 mL kg^−1^. Filled circles show an estimated treatment effect (Log diagnostic odds ratio) and its precision (standard error). In addition to individual study results, the fixed-effect estimates (vertical dashed line) with 95% confidence interval limits (diagonal dashed lines) and the random-effects estimate (vertical dotted line) are shown in the figure. The number of the point is the reference number of each study.

**Additional file 12: Table S2.** Other findings of meta-regression and subgroup analysis.

## Data Availability

The datasets used and/or analysed during the current study are available from the corresponding author on reasonable request.

## Abbreviations

Δ-IVC: The inferior vena cava respiratory variation
ABW: Actual body weight
APACHE II score: Acute Physiology, and Chronic Health Evaluation
ARDS: Acute respiratory distress syndrome
AUC: The area under curve
CI: Cardiac index
CO: Cardiac output
C-TD: Continuous thermodilution
C-PCA: Calibrated pulse contour analysis
DOR: Diagnostic odds ratio
EEOT: End-expiratory occlusion test
IBW: Ideal body weight
m-FC: Mini-fluid challenge
NC-PCA: Non-calibrated pulse contour analysis
NSS: Normal saline solution
PAC: Pulmonary artery catheter
PBW: Predicted body weight
PCA: Pulse contour analysis
PEEP: Positive end-expiratory pressure
PiCCO: Pulse contour cardiac output
PLR: Passive leg raising
PPV: Pulse pressure variation
PRAM: Pressure recording analytical method
TD: Thermodilution
TPTD: Transpulmonary thermodilution
TTE: Transthoracic echocardiographic
RL: Ringer’s lactate
ROC: Receiver operating characteristic
SOFA: Sequential Organ Failure Assessment
SV: Stroke volume
SVI: Stroke volume index
SVV: Stroke volume variation
TPTD: Transpulmonary thermodilution
Vt: Tidal volume
VtC: Tidal volume challenge
VTI: Velocity–time integral
