# Supplementary material for: Predictors of fluid responsiveness in critically ill patients mechanically ventilated at low tidal volumes: systematic review and meta-analysis
Source: Ann Intensive Care. 2021 Feb 8;11:28. doi: 10.1186/s13613-021-00817-5 (PMC7870741; doi:10.1186/s13613-021-00817-5)
Supplement: Supplementary file 1 — Additional file 1: Table S1. Additional. Risk of bias of the trials as assessed by QUADAS-2 criteria. [file 13613_2021_817_MOESM1_ESM.docx]

| **#** | **Study** | **Year** | **Patient selection** | **Index test** | **Reference Standard** | **Flow and timing** | **Patient selection** | **Index test** | **Reference Standard** | **Predictor to fluid responsiveness** |
| --- | --- | --- | --- | --- | --- | --- | --- | --- | --- | --- |
| 1 | De Backer (4) | 2005 | Low | Low | Low | Low | Low | Low | Low | PPV |
| 2 | Auler et al(26) | 2008 | Unclear | Low | Low | Low | Low | Low | Low | PPV |
| 3 | Huang et al (27) | 2008 | Unclear | Low | Low | Low | Low | Low | Low | PPV |
| 4 | Vistisen et al (28) | 2009 | Low | Low | Low | Low | Low | Low | Low | PPV |
| 5 | Vallée et al (29) | 2009 | Low | Low | Low | Low | Low | Low | Low | PPV |
| 6 | Muller et al (8) | 2010 | Unclear | Low | Low | Low | Low | Low | Low | PPV |
| 7 | Moretti et al (30) | 2010 | Low | Low | Low | Low | Low | Low | Low | ΔIVC |
| 8 | Lakhal et al (31) | 2011 | Low | Low | Low | Low | Low | Low | Low | PPV |
| 9 | Muller et al (32) | 2011 | Low | Low | Low | Low | Low | Low | Low | Mini-Fluid challenge |
| 10 | Monnet et al(7) | 2012 | Unclear | Low | Low | Low | Low | Low | Low | PPV. PLR. EEOT |
| 11 | Cecconi et al (33) | 2012 | Low | Low | Low | Low | Low | Low | Low | PPV. SVV |
| 12 | Yazigi et al (34) | 2012 | Low | Low | Low | Low | Low | Low | Low | PPV |
| 13 | Oliveira-Costa et al (35) | 2012 | Low | Low | Low | Low | Low | Low | Low | PPV |
| 14 | Drvar et al (36) | 2013 | High | Low | Low | Low | Low | Low | Low | PPV. SVV |
| 15 | Freitas et al (37) | 2013 | Low | Low | Low | Low | Low | Low | Low | PPV |
| 16 | Trepte et al(38) | 2013 | Unclear | Low | Low | Low | Low | Low | Low | PPV. SVV |
| 17 | Guarracino et al (39) | 2014 | Low | Low | Low | Low | Low | Low | Low | PPV |
| 18 | Kang et al (40) | 2014 | High | Low | Low | Low | Low | Low | Low | SVV |
| 19 | Ibarra-Estrada et al (41) | 2015 | Low | Low | Low | Low | Low | Low | Low | PPV. SVV. PLR |
| 20 | Angappan et al (42) | 2015 | High | Low | Low | Low | Low | Low | Low | SVV |
| 21 | Mallat et al (43) | 2015 | Unclear | Low | Low | Low | Low | Low | Low | Mini-Fluid challenge |
| 22 | Vistisen et al (44) | 2016 | Unclear | Low | Low | Low | Low | Low | Low | PPV |
| 23 | Liu et al (45) | 2016 | Low | Low | Low | Low | Low | Low | Low | PPV |
| 24 | Cherpanath et al (46) | 2016 | Unclear | Low | Low | Low | Low | Low | Low | PPV. SVV |
| 25 | Oliveira et al (47) | 2016 | Low | Low | Low | Low | Low | Low | Low | PPV. ΔIVC |
| 26 | Sobczyk et al (48) | 2016 | Low | Low | Low | Low | Low | Low | Low | PLR. ΔIVC |
| 27 | Myatra et al (49) | 2017 | Unclear | Low | Low | Low | Low | Low | Low | PPV. SVV. EEOT. Tidal volume challenge |
| 28 | Yonis et al (50) | 2017 | Low | Low | Low | Low | Low | Low | Low | PPV. Tidal volume challenge |
| 29 | Jozwiak et al (51) | 2017 | Low | Low | Low | Low | Low | Low | Low | EEOT |
| 30 | Guo-Guang Ma et al (52) | 2018 | Low | Low | Low | Low | Low | Low | Low | SVV. PLR. ΔIVC |
| 31 | Georges et al (53) | 2018 | High | Low | Low | Low | Low | Low | Low | EEOT |
| 32 | Depret et at (54) | 2019 | Unclear | Low | Low | Low | Low | Low | Low | EEOT |
| 33 | Fot et al (55) | 2019 | Unclear | Low | Low | Low | Low | Low | Low | Mini-Fluid challenge |

**Table 1 Additional. Risk of bias of the trials as assessed by QUADAS-2 criteria.** EEOT, end expiratory occlusion; PLR, passive leg raising; PPV, pression pulse variation; SVV, stroke volume variability; ΔIVC, inferior vena cava variability.
