# Supplementary figures and images for: Predictors of fluid responsiveness in critically ill patients mechanically ventilated at low tidal volumes: systematic review and meta-analysis
Source: Ann Intensive Care. 2021 Feb 8;11:28. doi: 10.1186/s13613-021-00817-5 (PMC7870741; doi:10.1186/s13613-021-00817-5)

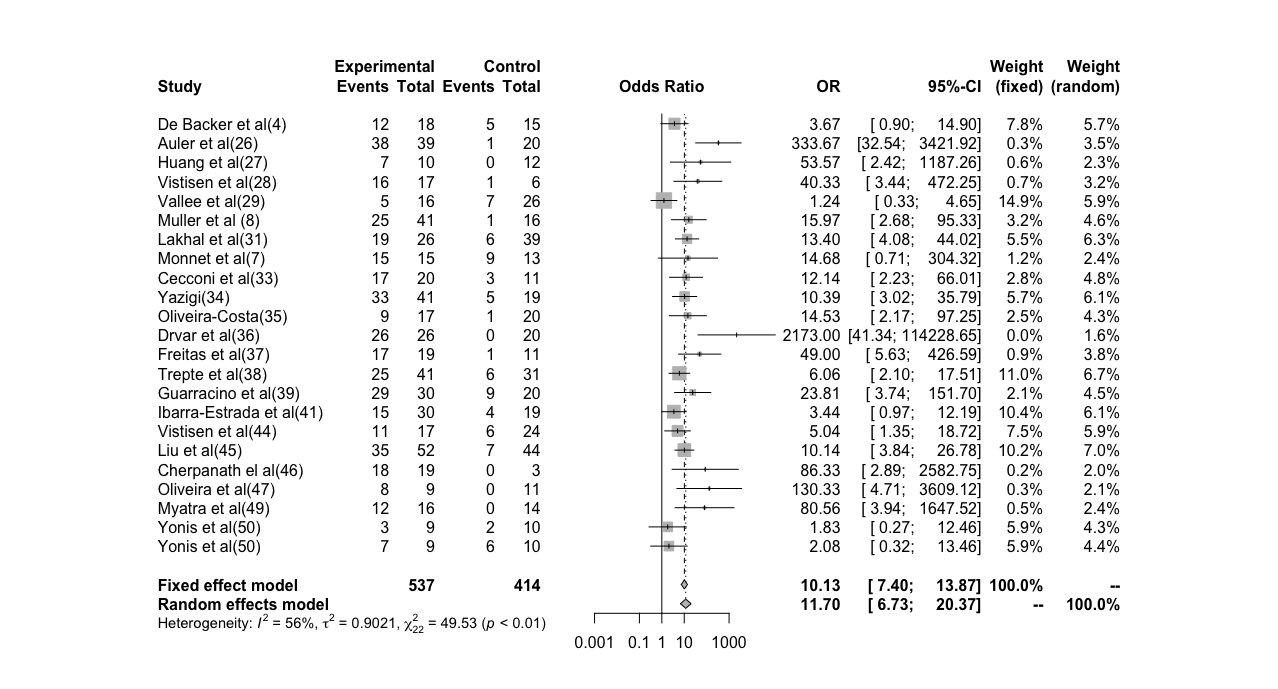

Supplement: Supplementary file 2 — Additional file 2: Figure S1. Diagnostic odds ratios of pulse pressure variation in adult critically ill ventilated patients with a Vt 8 ml kg−1 and without arrhythmia or respiratory effort. [file 13613_2021_817_MOESM2_ESM.tiff]

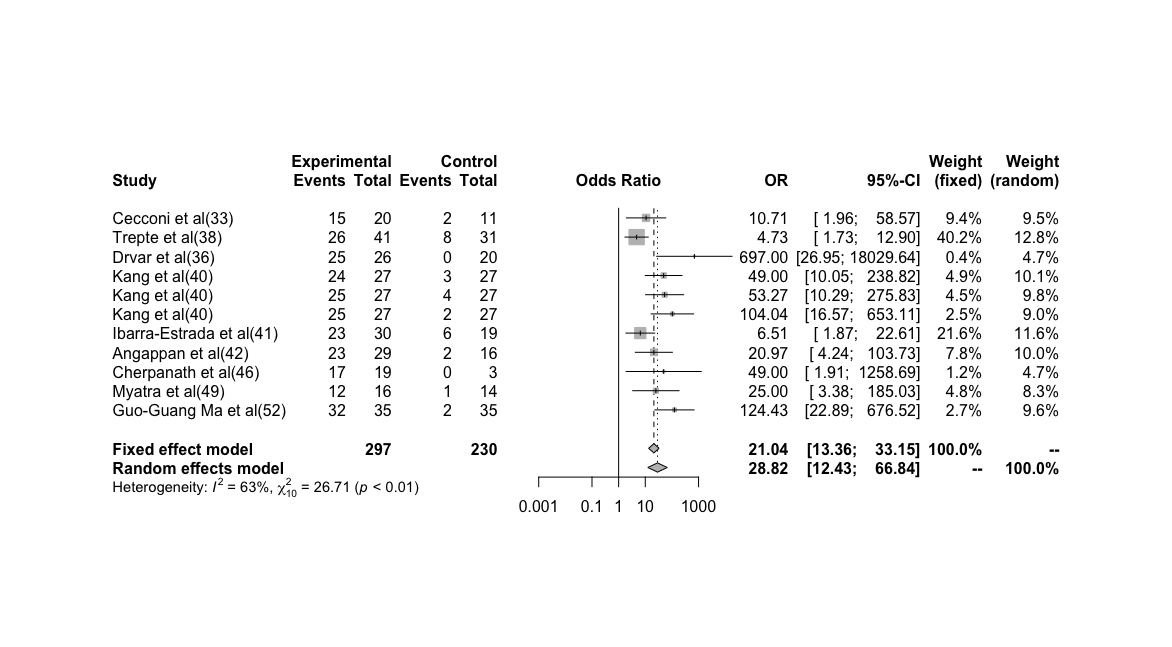

Supplement: Supplementary file 3 — Additional file 3: Figure S2. Diagnostic odds ratios of stroke volume variations in adult critically ill ventilated patients with a Vt 8 ml kg−1 and without arrhythmia or respiratory effort. [file 13613_2021_817_MOESM3_ESM.tiff]

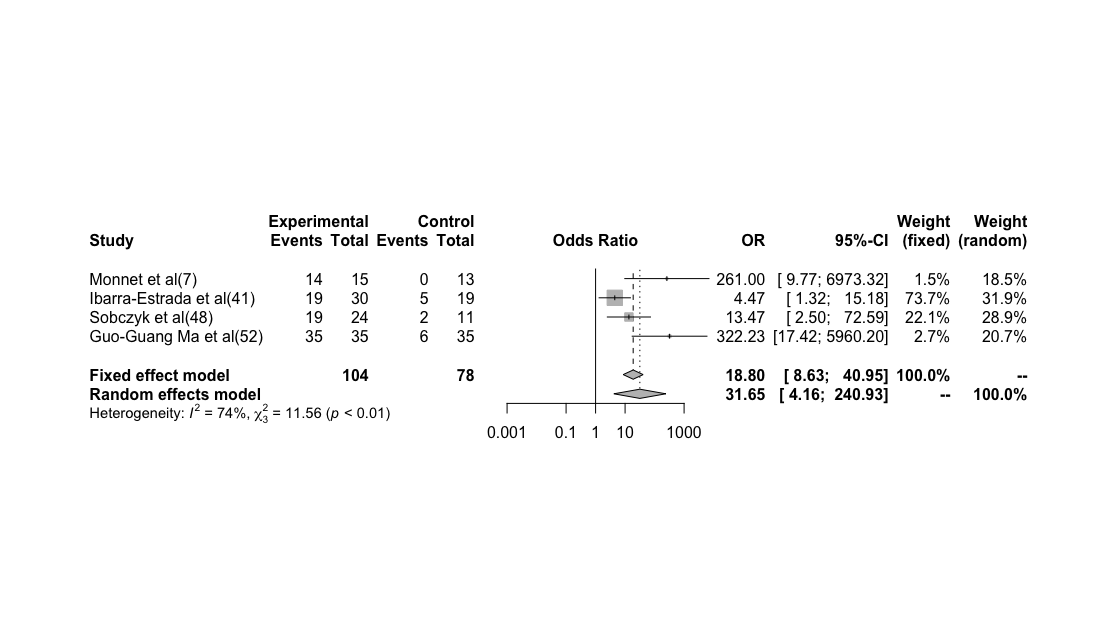

Supplement: Supplementary file 4 — Additional file 4: Figure S3. Diagnostic odds ratios of passive leg raising in adult critically ill ventilated patients with a Vt 8 ml kg−1 and without arrhythmia or respiratory effort. [file 13613_2021_817_MOESM4_ESM.tiff]

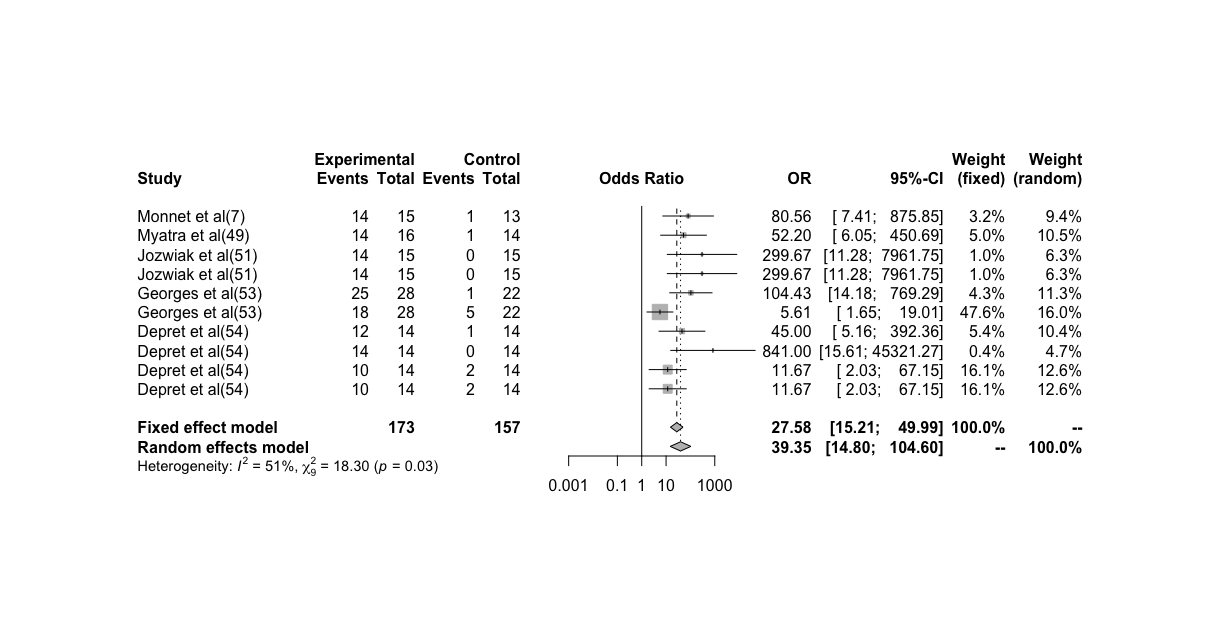

Supplement: Supplementary file 5 — Additional file 5: Figure S4. Diagnostic odds ratios of End-expiratory occlusion test in adult critically ill ventilated patients with a Vt 8 ml kg−1 and without arrhythmia or respiratory effort. [file 13613_2021_817_MOESM5_ESM.tiff]

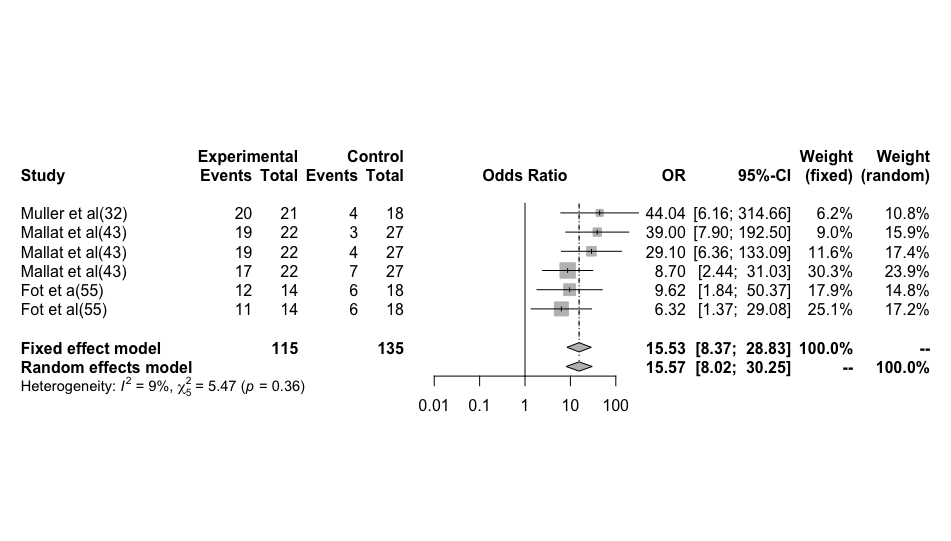

Supplement: Supplementary file 6 — Additional file 6: Figure S5. Diagnostic odds ratios of mini-fluid challenge in adult critically ill ventilated patients with a Vt 8 ml kg−1 and without arrhythmia or respiratory effort. [file 13613_2021_817_MOESM6_ESM.tiff]

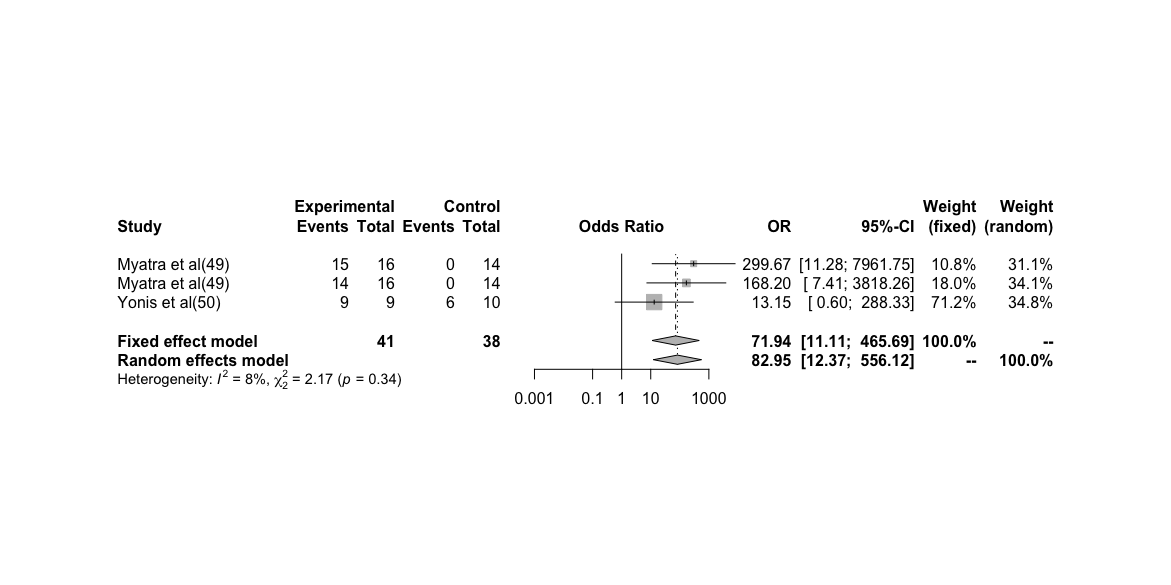

Supplement: Supplementary file 7 — Additional file 7: Figure S6. Diagnostic odds ratios of tidal volume challenge in adult critically ill ventilated patients with a Vt 8 ml kg−1 and without arrhythmia or respiratory effort. [file 13613_2021_817_MOESM7_ESM.tiff]

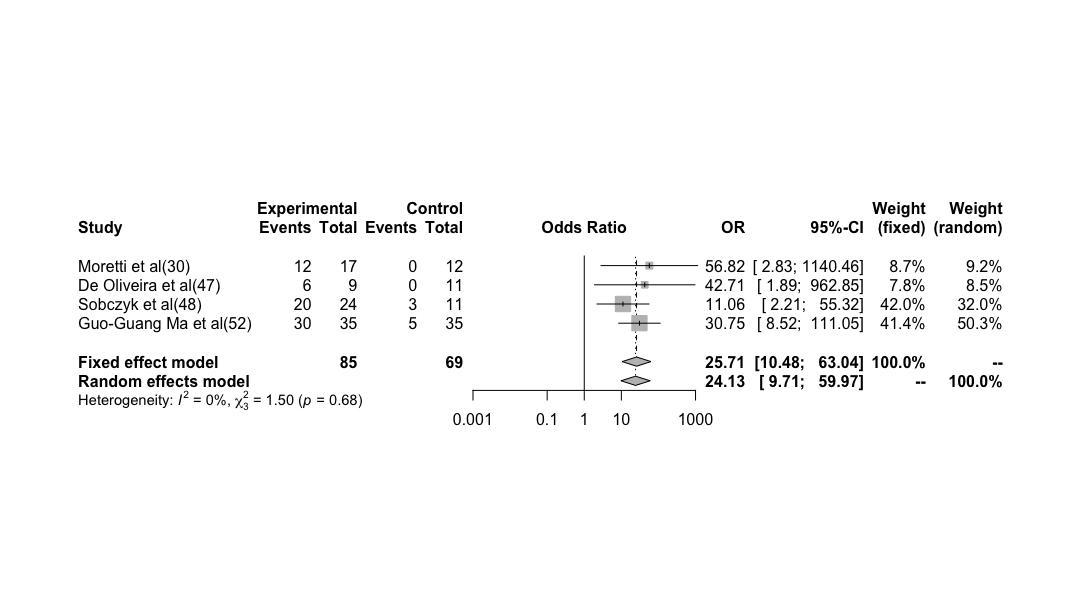

Supplement: Supplementary file 8 — Additional file 8: Figure S7. Diagnostic odds ratios of inferior vena cava respiratory variability in adult critically ill ventilated patients with a Vt 8 ml kg−1 and without arrhythmia or respiratory effort. [file 13613_2021_817_MOESM8_ESM.tiff]

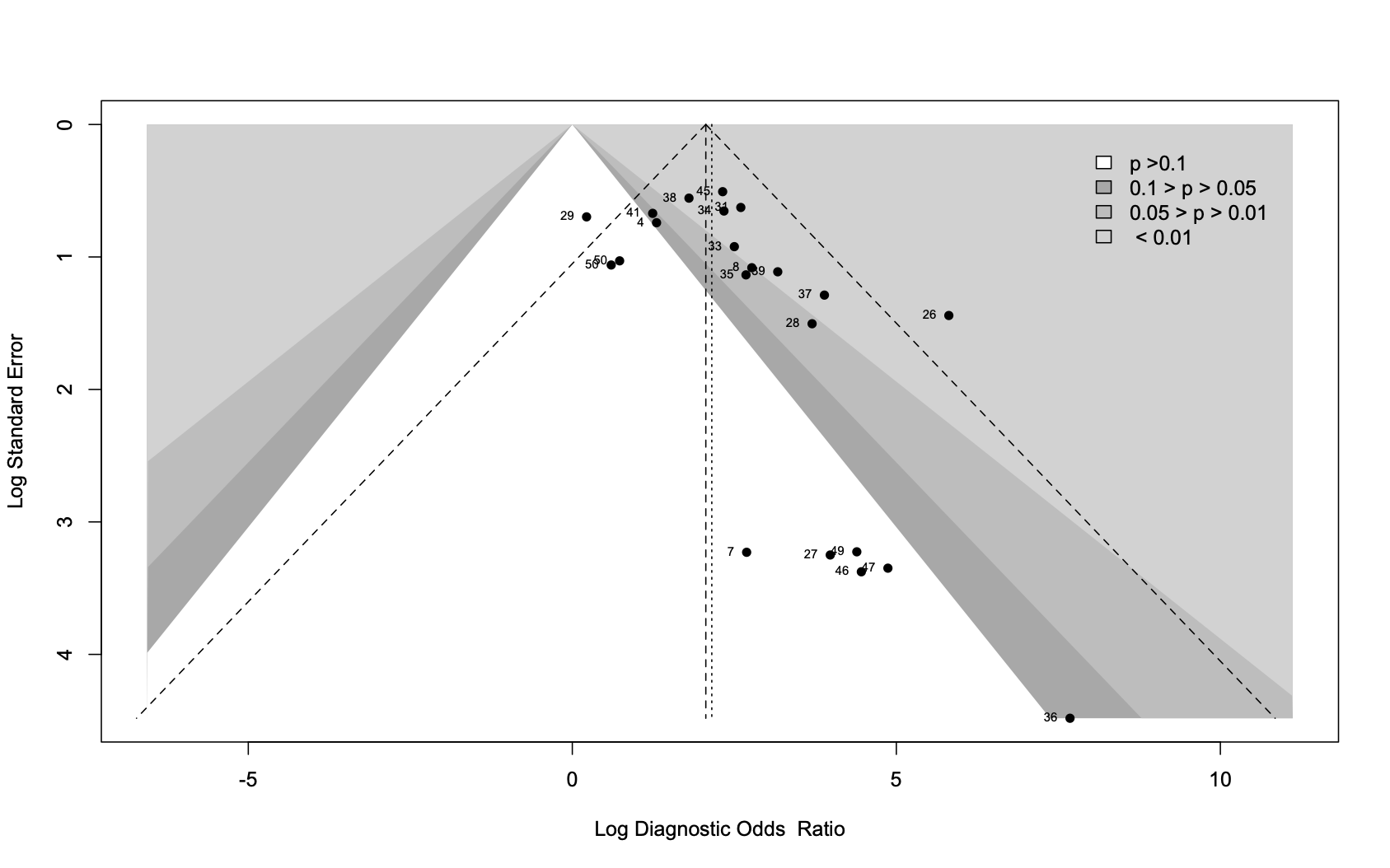

Supplement: Supplementary file 9 — Additional file 9: Figure S8. Contour enhanced funnel plot for a meta-analysis of pulse pressure variation for prediction of fluid responsiveness in patients with tidal volume 8 mL kg−1. Filled circles show an estimated treatment effect (Log diagnostic odds ratio) and its precision (standard error). In addition to individual study results, the fixed-effect estimates (vertical dashed line) with 95% confidence interval limits (diagonal dashed lines) and the random-effects estimate (vertical dotted line) are shown in the figure. The number of the point is the reference number of each study. [file 13613_2021_817_MOESM9_ESM.tiff]

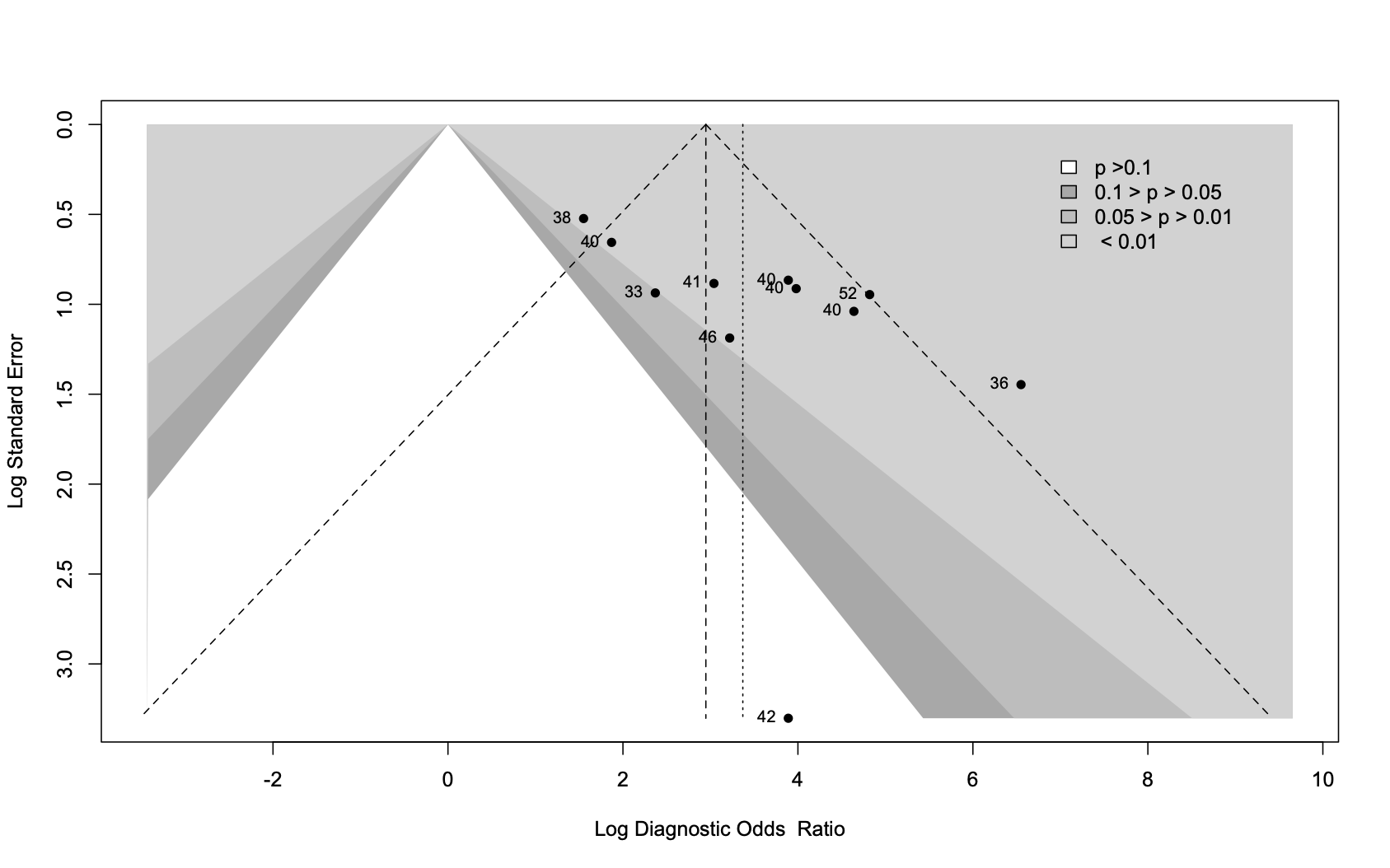

Supplement: Supplementary file 10 — Additional file 10: Figure S9. Contour enhanced funnel plot for a meta-analysis of stroke volume variation for prediction of fluid responsiveness in patients with tidal volume 8 mL kg−1. Filled circles show an estimated treatment effect (Log diagnostic odds ratio) and its precision (standard error). In addition to individual study results, the fixed-effect estimates (vertical dashed line) with 95% confidence interval limits (diagonal dashed lines) and the random-effects estimate (vertical dotted line) are shown in the figure. The number of the point is the reference number of each study. [file 13613_2021_817_MOESM10_ESM.tiff]

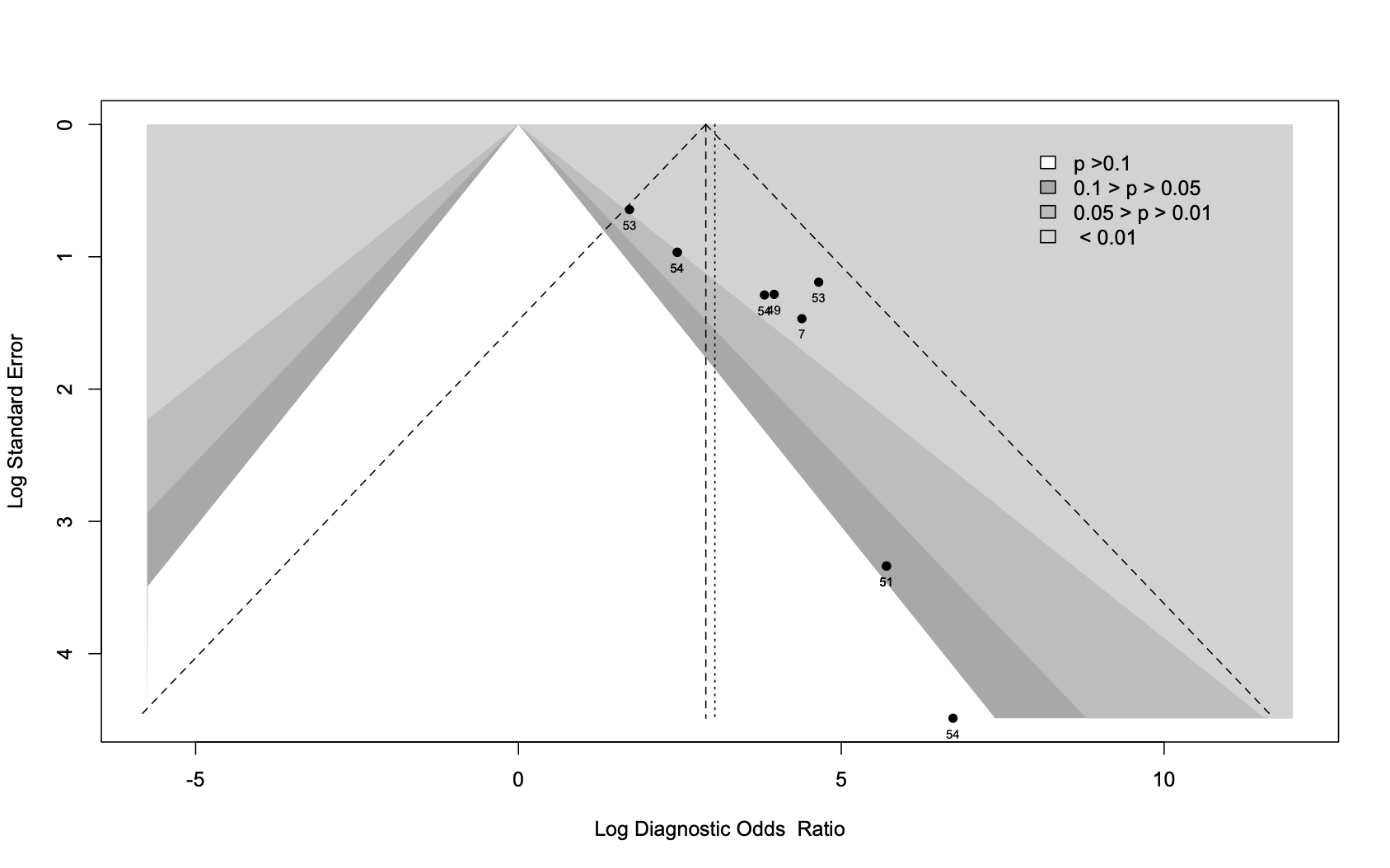

Supplement: Supplementary file 11 — Additional file 11: Figure S10. Contour enhanced funnel plot for a meta-analysis of end-expiratory occlusion test for prediction of fluid responsiveness in patients with tidal volume 8 mL kg−1. Filled circles show an estimated treatment effect (Log diagnostic odds ratio) and its precision (standard error). In addition to individual study results, the fixed-effect estimates (vertical dashed line) with 95% confidence interval limits (diagonal dashed lines) and the random-effects estimate (vertical dotted line) are shown in the figure. The number of the point is the reference number of each study. [file 13613_2021_817_MOESM11_ESM.tiff]
