# Supplementary material for: Predictors of fluid responsiveness in critically ill patients mechanically ventilated at low tidal volumes: systematic review and meta-analysis
Source: Ann Intensive Care. 2021 Feb 8;11:28. doi: 10.1186/s13613-021-00817-5 (PMC7870741; doi:10.1186/s13613-021-00817-5)
Supplement: Supplementary file 12 — Additional file 12: Table S2. Other findings of meta-regression and subgroup analysis. [file 13613_2021_817_MOESM12_ESM.docx]

| Subgroup | Predictor of intravenous fluid | Number of studies | Odds Ratio (95% IC) | P Value by meta-regression | P value by subgroup analysis | I^2^(%) | Q (value p) |
| --- | --- | --- | --- | --- | --- | --- | --- |
| Definition of fluid responsiveness | PPV |  |  |  |  |  |  |
| >15% |  | 21 | 14.67(7.63-28.20) | 0.66 | 0.66 | 56.94 | 48.77. p < 0.01 |
| >10% |  | 2 | 9.74(1.80-52.65) | <0.01 |  |  |  |
|  | EEOT | NA |  |  |  |  |  |
|  | PLR | NA |  |  |  |  |  |
|  | m-FC | NA |  |  |  |  |  |
|  | VtC | NA |  |  |  |  |  |
|  | Δ-IVC | NA |  |  |  |  |  |
| Critical care setting |  |  |  |  |  |  |  |
|  | PPV |  |  |  |  |  |  |
| Sepsis |  | 15 | 11.11(5.19-23.76) | <0.01 | 0.49 | 57.42 | 46.97. p <0.01 |
| Postsurgical |  | 3 | 14.74(2.84-76.62) | 0.76 |  |  |  |
| Cardiovascular |  | 5 | 28.51(7.29-111.48) | 0.24 |  |  |  |
|  | EEOT |  |  |  |  |  |  |
| Sepsis |  | 8 | 74.29(21.91-251.84) | <0.01 | 0.30 | 44.43 | 14.40. p =0.07 |
| Neurology |  | 2 | 22.17(3.05-161.39) | 0.30 |  |  |  |
|  | PLR |  |  |  |  |  |  |
| Sepsis |  | 2 | 24.06(0.83-695.80) | 0.06 | 0.69 | 74.13 | 7.73. p = 0.02 |
| Cardiovascular |  | 2 | 63.01(2.13-1867.06) | 0.69 |  |  |  |
|  | m-FC |  |  |  |  |  |  |
| Sepsis |  | 4 | 25.62(11.30-58.10) | <0.01 | 0.16 | 0.0 | 3.77. p = 0.44 |
| Cardiovascular |  | 2 | 9.14(2.76-30.24) | 0.16 |  |  |  |
|  | VtC | NA |  |  |  |  |  |
|  | Δ-IVC |  |  |  |  |  |  |
| Postsurgical |  | 2 | 36.97(10.81-126.51) | <0.01 | 0.57 | 0.0 | 0.01. p = 0.92 |
| Cardiovascular |  | 1 | 13.33(2.42-73.48) | 0.34 |  |  |  |
| Neurology |  | 1 | 56.82(2.83-1140.46) | 0.26 |  |  |  |
| Method used to measure the variable studied |  |  |  |  |  |  |  |
|  | PPV |  |  |  |  |  |  |
| Contour pulse wave analysis (PiCCO system) |  | 7 | 8.48(3.10-23.18) | <0.01 | 0.08 | 49.73 | 37.79. p <0.01 |
| Contour pulse wave analysis (LiDCO system) |  | 2 | 53.29(5.99-474.08) | 0.13 |  |  |  |
| Contour pulse wave analysis (computer software) |  | 4 | 68.56(13.27-354.23) | 0.03 |  |  |  |
| Analysis of arterial tracing |  | 10 | 9.81(4.20-21.36) | 0.82 |  |  |  |
|  | SVV |  |  |  |  |  |  |
| Contour pulse wave analysis (Vigileo) |  | 2 | 65.90(113.83-313.92) | <0.01 | 0.06 | 36.11% | 9.39. p = 0.15 |
| Contour pulse wave analysis (PiCCO system) |  | 3 | 8.14(2.68-24.71) | 0.03 |  |  |  |
| Contour pulse wave analysis (LiDCO system) |  | 2 | 42.60(6.44-281.69) | 0.73 |  |  |  |
| Contour pulse wave analysis (computer software) |  | 1 | 49.00(1.49-1611.02) | 0.88 |  |  |  |
| Bioreactance (NICOM system) |  | 3 | 86.12(23.62-313.95) | 0.80 |  |  |  |
|  | EEOT |  |  |  |  |  |  |
| Contour pulse wave analysis (PiCCO system) |  | 4 | 127.28(23.88-678.28) | <0.01 | 0.18 | 40.19 | 13.37. p = 0.10 |
| Others |  | 6 | 31.01(9.46-101.64) | 0.18 |  |  |  |
|  | PLR |  |  |  |  |  |  |
| Contour pulse wave analysis (Vigileo) |  | 1 | 322.23(1.04-99573.13) | 0.05 | 0.72 | 79.73% | 4.93. p = 0.03 |
| Contour pulse wave analysis (PiCCO system) |  | 2 | 26.31(0.55-1254.01) | 0.48 |  |  |  |
| TTE |  | 1 | 17.10(0.09-3294.48) | 0.46 |  |  |  |
|  | m-FC |  |  |  |  |  |  |
| Contour pulse wave analysis (PiCCO system) |  | 5 | 16.34(7.84-34.05) | <0.01 | 0.24 | 6.83 | 4.29. p = 0.37 |
| TTE |  | 1 | 70.00(6.77-723.32) | 0.24 |  |  |  |
|  | VtC | NA |  |  |  |  |  |
|  | Δ-IVC | NA |  |  |  |  |  |
| Volume of fluid challenge |  |  |  |  |  |  |  |
|  | PPV |  |  |  |  |  |  |
| 20 ml/kg |  | 1 | 722.00(25.25-20646.61) | <0.01 | 0.14 | 47.40 | 28.52. p = 0.02 |
| 250 ml |  | 1 | 15.11(1.17-194.40) | 0.07 |  |  |  |
| 250-500 ml |  | 1 | 23.44(1.45-379.23) | 0.12 |  |  |  |
| 300 ml |  | 1 | 6.51(0.79-53.62) | 0.02 |  |  |  |
| 500 ml |  | 11 | 14.74(6.22-34.62) | 0.03 |  |  |  |
| 500-1000 ml |  | 2 | 7.76(1.28-47.04) | 0.02 |  |  |  |
| 6 ml/kg |  | 1 | 1.23(0.13-11.87) | <0.01 |  |  |  |
| 7 ml/kg |  | 5 | 17.06(5.23-55.71) | 0.04 |  |  |  |
|  | EEOT |  |  |  |  |  |  |
| 500 ml |  | 9 | 53.61(16.60-173.08) | <0.01 | 0.78 | 54.81 | 17.70. p = 0.02 |
| 7 ml/kg |  | 1 | 91.00(2.63-3152.98) | 0.78 |  |  |  |
|  | m-FC |  |  |  |  |  |  |
| 500 ml |  | 4 | 25.62(11.30-58.10) | <0.01 | 0.16 | 0.0 | 3.77. p = 0.44 |
| 7 ml/kg |  | 2 | 9.14(2.76-30.24) | 0.16 |  |  |  |
|  | VtC |  |  |  |  |  |  |
| 500 ml |  | 1 | 13.15(0.60-288.33) | 0.10 | 0.14 | 0.0 | 0.06. p = 0.80 |
| 7 ml |  | 2 | 221.35(23.06-2124.25) | 0.15 |  |  |  |
|  | Δ-IVC |  |  |  |  |  |  |
| 250 ml |  | 1 | 13.33(2.42-73.48) | <0.01 | 0.56 | 0.0 | 0.01. p = 0.92 |
| 500 ml |  | 2 | 36.97(10.81-126.51) | 0.34 |  |  |  |
| 7 ml/kg |  | 1 | 56.82(2.83-1140.46) | 0.41 |  |  |  |
| Type of fluid used |  |  |  |  |  |  |  |
|  | PPV |  |  |  |  |  |  |
| NSS |  | 4 | 11.07(2.46-49.72) | <0.01 | 0.86 | 61.24% | 46.44. P <0.01 |
| LR and colloid |  | 1 | 23.44(1.04-530.73) | 0.67 |  |  |  |
| LR |  | 5 | 18.80(4.23-83.59) | 0.62 |  |  |  |
| LR and Colloid |  | 3 | 6.99(1.37-35.76) | 0.68 |  |  |  |
| Colloid |  | 10 | 17.80(6.68-47.47) | 0.60 |  |  |  |
|  | SVV |  |  |  |  |  |  |
| NSS |  | 2 | 14.08(1.66-119.54) | 0.02 | 0.33 | 64.60 | 25.43. p <0.01 |
| Colloid |  | 9 | 46.51(16.28-132.86) | 0.33 |  |  |  |
|  | EEOT | NA |  |  |  |  |  |
|  | PLR |  |  |  |  |  |  |
| NSS |  | 3 | 17.07)2.66-109.54) | <0.01 | 0.18 | 62.90 | 5.39. p=0.07 |
| Colloid |  | 1 | 322.23(6.87-15114.17) | 0.18 |  |  |  |
|  | m-FC |  |  |  |  |  |  |
| LR |  | 2 | 9.14(2.76-30.24) | <0.01 | 0.16 | 0.0 | 3.77. p = 0.44 |
| Colloid |  | 4 | 25.62(11.30-58.10) | 0.16 |  |  |  |
|  | VtC |  |  |  |  |  |  |
| NSS |  | 2 | 221.35(23.06-2124.25) | <0.01 | 0.15 | 0.0 | 0.06. p = 0.80 |
| LR |  | 1 | 13.15(0.60-288.33) | 0.15 |  |  |  |
|  | Δ-IVC |  |  |  |  |  |  |
| NSS |  | 1 | 13.33(2.42-73.48) | <0.01 | 0.59 | 0.0 | 0.07; p = 0.79 |
| LR |  | 1 | 1.89-962.85) | 0.52 |  |  |  |
| Colloid |  | 2 | 38.84(11.44-131.89) | 0.32 |  |  |  |
| Variable used to determinate fluid responsiveness |  |  |  |  |  |  |  |
|  | PPV |  |  |  |  |  |  |
| CO |  | 4 | 23.27(5.34-101.35) | <0.01 | 0.82 | 60.41 | 42.94. p <0.01 |
| CI |  | 12 | 11.27(4.55-27.95) | 0.41 |  |  |  |
| CI and SVI |  | 1 | 23.44(1.06-520.10) | 1.00 |  |  |  |
| SV |  | 2 | 8.70(1.24-61.15) | 0.43 |  |  |  |
| SVI |  | 3 | 14.91(2.70-82.38) | 0.70 |  |  |  |
| VTI |  | 1 | 130.33(2.34-7247.24) | 0.43 |  |  |  |
|  | SVV |  |  |  |  |  |  |
| CO |  | 4 | 80.15(18.33-350.40) | <0.01 | 0.43 | 53.99 | 15.22. p = 0.03 |
| CI |  | 3 | 14.04(3.05-64.73) | 0.11 |  |  |  |
| SV |  | 2 | 48.48(6.83-344.15) | 0.69 |  |  |  |
| SVI |  | 2 | 26.32(3.36-206.24) | 0.39 |  |  |  |
|  | EEOT |  |  |  |  |  |  |
| CO |  | 2 | 22.17(3.05-161.39) | <0.01 | 0.31 | 44.43 | 14.40. p = 0.07 |
| CI |  | 8 | 74.29(21.91-251.84) | 0.31 |  |  |  |
|  | PLR | NA |  |  |  |  |  |
|  | m-FC |  |  |  |  |  |  |
| CI |  | 5 | 16.34(7.84-34.05) | <0.01 | 0.24 | 6.83 | 4.29. p = 0.37 |
| VTI |  | 1 | 70.00(6.77-723.32) | 0.24 |  |  |  |
|  | VtC | NA |  |  |  |  |  |
|  | Δ-IVC | NA |  |  |  |  |  |
| Sensitivity analysis |  |  |  |  |  |  |  |
|  | PPV |  |  |  |  |  |  |
| Low risk |  | 17 | 11.69(5.83-23.41) | <0.01 | 0.34 | 56.44 | 48.21. p <0.01 |
| Unclear risk |  | 6 | 22.68(6.81-75.57) | 0.34 |  |  |  |
|  | SVV |  |  |  |  |  |  |
| Low risk |  | 6 | 21.61(7.34-63.59) | <0.01 | 0.35 | 57.21 | 18.70. p = 0.02 |
| High risk |  | 3 | 87.12(18.20-417.37) | 0.15 |  |  |  |
| Unclear risk |  | 2 | 42.66(3.91-465.22) | 0.61 |  |  |  |
|  | EEOT |  |  |  |  |  |  |
| Low |  | 8 | 74.21(21.91-251.84) | <0.01 | 0.31 | 44.43% | 14.40. p = 0.07 |
| High |  | 2 | 22.17(3.05-161.39) | 0.31 |  |  |  |
|  | PLR | NA |  |  |  |  |  |
|  | m-FC | NA |  |  |  |  |  |
|  | VtC | NA |  |  |  |  |  |
|  | Δ-IVC |  |  |  |  |  |  |
| Low |  | 3 | 27.03(10.00-73.02) | <0.01 | 0.78 | 0.0 | 1.07. p = 0.59 |
| Unclear |  | 1 | 42.71(1.89-962.85) | 0.78 |  |  |  |
| Tidal volume |  |  |  |  |  |  |  |
|  | PPV | 23 | DOR = 2.69 + 1.02 IC 95% 0.65-1.58) *Vt (ml/kg) | 0.93 | NA | 53.91 | 45.56. p <0.01 |
|  | SVV | 11 | DOR = 6.49 + 0.98 (IC 95% 0.58-1.64) * Vt(ml/kg) | 0.95 | NA | 47.46 | 17.13. p = 0.05 |
|  | EEOT | 10 | DOR = 6.95 + 1.02 (IC 95% 0.42-2.43) | 0.97 | NA | 21.62 | 10.21. p = 0.25 |
|  | PLR | 4 | DOR = 0.27 + 1.44 (IC 95% 0.87-2.41) * Vt (ml/kg) | 0.15 | NA | 0.0 | 1.96. p = 0.38 |
|  | m-FC | 6 | DOR = 354.24 + 0.52(IC 95% 0.24- 1.11) * Vt(ml/kg) | 0.09 | NA | 0.0 | 2.82. p = 0.59 |
|  | VtC | NA |  |  |  |  |  |
|  | Δ-IVC | 4 | DOR = 5.15(2.82-9.39) | 0.45 | NA | 0.0 | 2.65. p = 0.45 |
| PEEP |  |  |  |  |  |  |  |
|  | PPV | 21 | DOR = 3.63 + 0.97 (IC 95% 0.86-1.10) + PEEP (mmHg) | 0.69 | NA | 54.04 | 41.34. p <0.01 |
|  | SVV | 10 | DOR = 5.15 + 1.02 (IC 95% 0.81- 1.28) + PEEP (mmHg) | 0.84 | NA | 52.85 | 16.97. p = 0.03 |
|  | EEOT | 10 | DOR = 2.82 + 1.11 (IC 95% 0.87-1.41) + PEEP (mmHg) | 0.39 | NA | 10.14% | 8.90. p = 0.35 |
|  | PLR | 4 | DOR = 2.38 + 1.11 (IC 95% 0.32-3.85) + PEEP (mmHg) | 0.86 | NA | 46.00 | 3.70. p = 0.16 |
|  | m-FC | 6 | DOR = 1.36 + 1.16 (IC 95% 0.93-1.43) * + PEEP (mmHg) | 0.19 | NA | 0.00 | 3.90. p = 0.42 |
|  | VtC | NA |  |  |  |  |  |
|  | Δ-IVC | 4 | DOR = 17.81 + 0.77 (IC 95% 0.44-1.34) | 0.36 | NA | 0.00 | 1.81. p = 0.40 |
| Driving pressure |  |  |  |  |  |  |  |
|  | PPV | 12 | DOR = 9.77 + 0.91 (IC 95% 0.79-1.04) * driving pressure (mmHg) | 0.17 | NA | 47.49 | 19.04. p = 0.04 |
|  | SVV | NA |  |  |  |  |  |
|  | EEOT | 10 | DOR = 1.44 + 1.15 (IC 95% 0.90-1.44) * driving pressure (mmHg) | 0.25 | NA | 6.72 | 8.58. p = 0.38 |
|  | PLR | NA |  |  |  |  |  |
|  | m-FC | 4 | DOR = 2.80 + 1.04 (IC 95% 0.54-1.97) * driving pressure (mmHg) | 0.91 | NA | 25.89 | 2.70. p = 0.26 |
|  | VtC | NA |  |  |  |  |  |
|  | Δ-IVC | NA |  |  |  |  |  |
| Lung Compliance |  |  |  |  |  |  |  |
|  | SVV | NA |  |  |  |  |  |
|  | EEOT | 10 | DOR = 59.73 + 0.95 (IC 95% 0.88-1.01) * Compliance (ml/mmHg) | 0.08 | NA | º0.0 | 7.26. p = 0.51 |
|  | PLR | NA |  |  |  |  |  |
|  | m-FC | 4 | DOR = 2.82 + 1.01 (IC 95% 0.77-1.33) * Compliance (ml/mmHg) | 0.91 | NA | 25.89 | 2.70. p = 0.26 |
|  | VtC | NA |  |  |  |  |  |
|  | Δ-IVC | NA |  |  |  |  |  |

**Table 2 Additional.** **Other findings of meta-regression and subgroup analysis**. CI, cardiac index; CO, cardiac output; I^2^, inconsistency; TTE, EEOTT, end expiratory occlusion; LR. lactate ringer; m-FC, mini-fluid challenge; NA, not applied; NSS, normal saline solution; PEEP, positive end-expiratory pressure; PiCCO, Pulse contour cardiac output; PLR, passive leg raising; PPV, pression pulse variation; SVV, stroke volume variability; IVC, inferior vena cava variability.; PPV, pulse pressure variation; PLR, passive leg raising; Q, Cochrane statistics; SV, stroke volume; SVI, stroke volume index; SVV, stroke volume variability; VtC, tidal volume challenge; VTI, velocity time integral; TTE, transthoracic echocardiography; Values are expressed as pooled data (95% confidence interval).
